# The epigenetic modifier HDAC2 and the checkpoint kinase ATM determine the responses of microsatellite instable colorectal cancer cells to 5-fluorouracil

**DOI:** 10.1007/s10565-022-09731-3

**Published:** 2022-05-24

**Authors:** Nicole Kiweler, Helena Schwarz, Alexandra Nguyen, Stephanie Matschos, Christina Mullins, Andrea Piée-Staffa, Christina Brachetti, Wynand P. Roos, Günter Schneider, Michael Linnebacher, Walburgis Brenner, Oliver H. Krämer

**Affiliations:** 1 Department of Toxicology, University Medical Center Mainz, 55131 Mainz, Germany; 2 Present Address: Department of Cancer Research, Luxembourg Institute of Health, L-1526 Luxembourg, Luxembourg; 3 Department of General Surgery, Molecular Oncology and Immunotherapy, Schillingallee 35, 18057 Rostock, Germany; 4 Klinikum Rechts Der Isar, Medical Clinic and Polyclinic II, Technical University Munich, 81675 Munich, Germany; 5 Department of General, Visceral and Pediatric Surgery, University Medical Center Göttingen, 37075 Göttingen, Germany; 6 Clinic for Obstetrics and Women’s Health, Johannes Gutenberg University Mainz, Mainz, Germany

**Keywords:** 5-FU, ATM, Clonal evolution, HDAC2, KAP1, KU-60019, Histone acetylation, PR130, DNA replication stress, Tumor heterogeneity

## Abstract

The epigenetic modifier histone deacetylase-2 (HDAC2) is frequently dysregulated in colon cancer cells. Microsatellite instability (MSI), an unfaithful replication of DNA at nucleotide repeats, occurs in about 15% of human colon tumors. MSI promotes a genetic frameshift and consequently a loss of HDAC2 in up to 43% of these tumors. We show that long-term and short-term cultures of colorectal cancers with MSI contain subpopulations of cells lacking HDAC2. These can be isolated as single cell-derived, proliferating populations. Xenografted patient-derived colon cancer tissues with MSI also show variable patterns of HDAC2 expression in mice. HDAC2-positive and HDAC2-negative RKO cells respond similarly to pharmacological inhibitors of the class I HDACs HDAC1/HDAC2/HDAC3. In contrast to this similarity, HDAC2-negative and HDAC2-positive RKO cells undergo differential cell cycle arrest and apoptosis induction in response to the frequently used chemotherapeutic 5-fluorouracil, which becomes incorporated into and damages RNA and DNA. 5-fluorouracil causes an enrichment of HDAC2-negative RKO cells in vitro and in a subset of primary colorectal tumors in mice. 5-fluorouracil induces the phosphorylation of KAP1, a target of the checkpoint kinase ataxia-telangiectasia mutated (ATM), stronger in HDAC2-negative cells than in their HDAC2-positive counterparts. Pharmacological inhibition of ATM sensitizes RKO cells to cytotoxic effects of 5-fluorouracil. These findings demonstrate that HDAC2 and ATM modulate the responses of colorectal cancer cells towards 5-FU.

**Graphical abstract:**

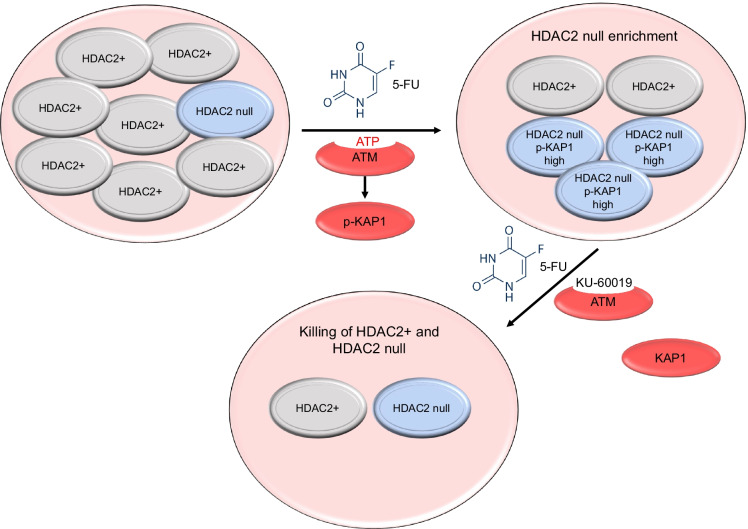

**Supplementary Information:**

The online version contains supplementary material available at 10.1007/s10565-022-09731-3.

## Highlights

- HDAC2 status determines responses of colon cancer cells to 5-FU and activation of the ATM-KAP1 signaling axis.
- Enrichment of HDAC2 null cells can occur during drug-induced DNA replication stress/DNA damage.
- ATM is a druggable vulnerability in cancer cells exposed to 5-FU.

**Supplementary Information:**

The online version contains supplementary material available at 10.1007/s10565-022-09731-3.

## Introduction

With about 1.8 million new cases every year, colon cancer is the third most diagnosed malignancy and the second to fourth leading cause of cancer-related deaths worldwide (Jung et al. 2020; Keum and Giovannucci 2019; Neitzel et al. 2020; Oneda and Zaniboni 2021; Patnaik and Anupriya 2019). Genetic predisposition, inflammatory bowel diseases, and environmental factors such as lifestyle (smoking, alcohol, red meat consumption, etc.) can promote colorectal tumorigenesis (Neitzel et al. 2020; Seiwert et al. 2020). This disease evolves from a largely defined, stepwise sequence of genetic and early occurring epigenetic aberrations. These transform normal colon epithelial cells to adenomas and invasive adenocarcinomas (Jung et al. 2020; Keum and Giovannucci 2019). Up to 30% of colorectal cancers arise from hyperplastic and serrated polyps (De Palma et al. 2019). Inflammation-associated colorectal tumors are in the low percentage range (Keller et al. 2019).

Limitations to successful colorectal cancer therapy are the late detection of the disease in about half of the cases and the recurrence and metastatic progression of the disease. Moreover, therapy-resistant colon cancer cells can be present within the primary tumor or they can arise as escape mutants of chemotherapy (De Palma et al. 2019; Jung et al. 2020; Keum and Giovannucci 2019; Neitzel et al. 2020). Treatment regimen for colorectal cancer frequently include the anti-metabolite 5-fluorouracil (5-FU), which can be given as its orally available prodrug capecitabine (Neitzel et al. 2020; Oneda and Zaniboni 2021; Peters et al. 2002; Vodenkova et al. 2020; Wilson et al. 2014; Wyatt and Wilson 2009). This drug is taken up by cells and metabolized to 5-fluoro-UTP and 5-fluoro-dUTP. These metabolites are incorporated into and subsequently damage RNA and DNA, respectively. Suicide inhibition of thymidylate synthase (TS) by the 5-FU metabolite 5-fluoro-dUMP fosters the incorporation of 5-fluoro-dUTP over the endogenous DNA building block dTTP (Neitzel et al. 2020; Peters et al. 2002; Vodenkova et al. 2020). Incorporated 5-FdUTP causes a complex DNA damage response consisting of nucleotide mismatches in the S phase, futile cycles of base excision repair to remove the mis-incorporated fluoropyrimidines, and the subsequent loss of functional RNAs and proteins. These lesions are cytotoxic (Wilson et al. 2014; Wyatt and Wilson 2009).

Among the epigenetic factors that control the development and fate of colon cells are the 18 histone deacetylases (HDACs) (Jung et al. 2020; Patnaik and Anupriya 2019). HDAC2 belongs to the group of class I HDACs (HDAC1,-2,-3,-8) and is frequently dysregulated in colon cancer cells (Krämer 2009; Wagner et al. 2014). Both overexpression and loss of HDAC2 are observed. Zhu and colleagues reported that HDAC2 was overexpressed in colon tumor explants and that the oncogenes APC and MYC controlled HDAC2 expression (Zhu et al. 2004). Inhibition of class I HDACs with the histone deacetylase inhibitor (HDACi) valproic acid (VPA) and a consequently accelerated proteasomal degradation of HDAC2 (Krämer et al. 2003) could block colonic adenoma formation in mice (Zhu et al. 2004), underscoring the relevance of HDAC2.

Microsatellites are repeats of nucleotides, such as (A)n or (CA)n that are dispersed throughout mammalian genomes (Vilar and Gruber 2010). About 15% of colorectal cancers have an unfaithful replication of microsatellites (microsatellite instability, MSI). This stems from mutations in key DNA mismatch repair pathway genes, including epigenetic silencing of MLH1 and inherited mutations in MLH1, MSH2, MSH6, and PMS2. The gene encoding HDAC2 has an AT-rich sequence containing 9 adenosine residues in its first exon and 17 (Ropero et al. 2006) to 43% (Hanigan et al. 2008) of colon tumors with MSI carry mutations in the gene encoding HDAC2. This was not seen in colon tumor cells with stable microsatellites (Hanigan et al. 2008; Ropero et al. 2006). Endometrial and gastric tumors with MSI also have HDAC2 mutated in about 19–29% of cases (Ropero et al. 2006).

Ropero and colleagues reported that RKO cells (from colorectal cancer with MSI) were negative for HDAC2 and insensitive to the HDACi trichostatin-A, which can block all 11 zinc-dependent HDACs (Ropero et al. 2006). However, Ree and colleagues showed that RKO cells from the American Type Culture Collection (ATCC) expressed HDAC2 and that RKO cells responded to TSA irrespective of HDAC2 expression (Ree et al. 2008). Hanigan and colleagues confirmed the existence of HDAC2-negative colorectal cancer cells and that such cells occur in primary human colorectal cancer tissues (Hanigan et al. 2008). Conflicting data on how one cell line can be classified as HDAC2-positive or HDAC2-negative in different laboratories and the uncertainty whether the HDAC2 status determines the cellular responsiveness to HDACi indicates the need for further research. It is likewise unclear whether HDAC2 determines cellular responses to chemotherapies, such as 5-FU.

DNA replication stress and DNA damage activate checkpoint kinases. These enzymes ensure cell cycle arrest, DNA repair, and also cell death upon irreparable DNA damage (Dobbelstein and Sørensen 2015). MSI can be associated with altered checkpoint kinase signaling in colorectal cancers. The nucleolytic DNA double-strand break repair protein MRE11A initiates checkpoint kinase signaling through the checkpoint kinase ATM. MRE11A has a genomic poly-T(11) repeat and a loss of MRE11A, which occurs in 70–85% of MSI cases, might be associated with the drug sensitivity of MSI colorectal cancer cells. Furthermore, the DNA repair protein RAD50 interacts with MRE11A and can become mutated in MSI cancers upon the unfaithful duplication of a poly-A(9) repeat (Miquel et al. 2007; Vilar and Gruber 2010). MSI colorectal cancer cells with a loss of the checkpoint kinases ATR, DNA-PK, and CHK1 were also found. These mutations disengage cell cycle control and DNA repair in cells with DNA replication stress and DNA damage (Lewis et al. 2007; Miquel et al. 2007).

HDACi decrease DNA repair proteins in tumor cells, and this increases the toxicity of chemotherapies that cause DNA replication stress and DNA damage (Göder et al. 2018; Kiweler et al. 2018; Nikolova et al. 2017). It is likewise established that 5-FU induces a class I HDAC-dependent activation of checkpoint kinases in MSI colon cancer cells (Göder et al. 2018). However, it is unknown whether the HDAC2 status of MSI cancers is linked to the expression levels of checkpoint kinases and enzymes that produce nucleotides and metabolize 5-FU, such as TS or ribonucleotide reductase (RNR). Their expression determines sensitivities of tumor cells towards 5-FU (Fukushima et al. 2001; Vodenkova et al. 2020).

Despite the clear evidence that colon cancers with MSI can exist in HDAC2-proficient and HDAC2-deficient states, surprisingly little is known about the molecular consequences. We hypothesized that HDAC2-negative cells were a subpopulation of cells with unique properties. Moreover, we speculated that HDAC2-positive and HDAC2-negative cells differentially responded to HDACi-induced protein hyperacetylation and to DNA replication stress induction by 5-FU.

## Materials and methods

### Cell lines

HDAC2-positive RKO cells (RKO^HDAC2^) were originally from the DSMZ Braunschweig, Germany, and provided by Dr. M. Zörnig, GSH Frankfurt/Main, Germany (Krämer et al. 2008). RKO^ΔHDAC2^ cells were provided by Prof. M. Esteller, Idibell Barcelona, Spain (Ropero et al. 2006). HROC cells are primary MSI colorectal cancers (Maletzki et al. 2012). All cells were cultured in Dulbecco’s Modified Eagle’s Medium (DMEM; GE Healthcare, UK) supplemented with 5% (RKO cells) or 10% fetal calf serum (HROC cells) (Sigma-Aldrich, Munich, or Lonza, Cologne, Germany), 100 U/mL penicillin, and 100 µg/mL streptomycin (Sigma-Aldrich, Munich, Germany).

### Animal experiments and HDAC2 immunohistochemistry

We analyzed patient-derived xenografts (PDXs) using a 1 × 1 × 1 experimental design, termed PDX clinical trial (Gao et al. 2015). Due to the high engraftment rate of up to 80%, the preferred mice strain was NOD.Cg-Prkdcscid Il2rgtm1Wjl/SzJ (NSG). Mice were bred in the animal facility of the Rostock University Medical Center and maintained in pathogen-free conditions and exposed to 12-h light/12-h darkness cycles. During engraftment and therapy, mice received standard pellet food and water ad libitum. Before xenografting, vital HROC PDX tumor aliquots (3 × 3 × 3 mm) were soaked in 100 µL Matrigel (Corning, Kaiserslautern, Germany) for > 10 min at 4 °C. Afterward, these tumor pieces were implanted subcutaneously into the animals’ right flank under anesthesia (ketamine/xylazine, 90/6 mg/kg body weight). Tumor growth was subsequently monitored at least weekly. Therapy with 5-FU was initiated upon tumor establishment of ~ 6 mm diameter for 18 days with a dose of 20 mg/kg body weight intraperitoneally thrice weekly. The same procedure was done for the control group with an injection of 100 µL sodium chloride. After tumor resection, one part of the PDX tumor was fixed immediately in formalin and embedded in paraffin by routine procedures. For immunohistochemical staining, purified anti-HDAC2 (clone 3F3/HDAC2; BioLegend, San Diego, USA) was used in a dilution of 1:100 in PBS overnight. Labeled polymer-HRP anti-mouse (Envision Kit, Agilent-Dako, Santa Clara, USA) was used as secondary antibody according to the manufacturer’s protocol (gives brown stain indicating HDAC2 positivity). Slides were counterstained with Mayer’s hematoxylin (blue stain). HDAC2-stained sections (4–5 μm) were taken for light-microscopic study to assess HDAC2 in the PDX models.

### Drugs and antibodies

5-FU, oxaliplatin (L-OHP), and propidium iodine (PI) were purchased from Sigma-Aldrich, Munich, Germany; MS-275 and MERCK60 were from Selleck Chemicals, Munich, Germany; and TO-PRO-3 was from Life Technologies, Ober-Olm, Germany. Antibodies for immunoblot were from: Millipore, Darmstadt, Germany (HDAC1 (05–100); acetylated histone H3 (06–599)), Santa Cruz Biotechnology, Heidelberg, Germany (HDAC2 (sc-7899); ɣH2AX (sc-101696)), Enzo Life Sciences, Lörrach, Germany (HSP90 (ADI-SPA-830)), Novus Biologicals, Heidelberg, Germany (p-KAP1 (Ser824), NB100-2350), Abcam, Cambridge, UK (p-ATM (Ser1981), ab81292; HDAC3, ab16047), Cell Signaling Technology, Frankfurt/Main, Germany (ATM (cs-2873); p-AKT1/p-AKT2/p-AKT3 (cs-9271; Ser473); CHK1 (cs-2360); HDAC6 (cs-7558); anti-p-Ser15-p53 (cs-9284); TS (cs-9045)), Sigma-Aldrich, Munich, Germany (acetylated tubulin (6-11B-1), T7451), BD Biosciences, Heidelberg, Germany (β-catenin (BD610154)), Thermo Fisher Scientific, Frankfurt/Main, Germany (RRM2 (PA5-13,570); vimentin (V9), MS-129-P). Antibodies against HDAC2 (#5113) from Cell Signaling Technology, Frankfurt/Main, Germany, and HDAC1 (05–100) from Millipore, Darmstadt, Germany, were used for immunofluorescence analysis.

### Protein lysate preparation and immunoblot

We previously described these methods in Beyer et al. (2017).

### Cell cycle and cell death analysis by flow cytometry using PI

We recently described this method in Beyer et al. (2022).

### Immunofluorescence analysis

Cells were seeded on coverslips for 24 h. Coverslips were washed twice with PBS and cells were fixed and permeabilized with ice-cold (− 20 °C) methanol:acetone (7:3) for 8 min. To remove remaining fixative, coverslips were washed thrice with PBS before incubation with blocking solution (5% bovine serum albumin (BSA), 0.3% Triton X-100 in PBS) for 1 h. Cells were incubated with HDAC2 (diluted 1:400) or HDAC1 (diluted 1:200) primary antibody in PBS (supplemented with 1% BSA, 0.3% Triton X-10) overnight at 4 °C. Coverslips were washed three times with PBS and incubated with the appropriate AF488-conjugated secondary antibody (diluted 1:400 in PBS/0.3% Triton X-100) for 3 h at RT; antibodies were from Santa Cruz, Heidelberg, Germany. Following three washing steps (PBS), the nuclear staining was performed for 15 min using TO-PRO-3 (1 µM in PBS). Slides were mounted with Vectashield® (Vector Labs, Bath, UK). Analysis and image capturing were performed via confocal microscopy using a Zeiss Axio Observer.Z1 microscope equipped with an LSM710 laser-scanning unit (Zeiss, Jena, Germany).

### Long-term treatment with 5-FU/L-OHP

RKO^HDAC2^ cells were cultivated for 12 days. The culture medium was changed and treatment with 2 µM 5-FU or 2 µM L-OHP was applied every second day. For assessment of HDAC2 expression, immunofluorescence analysis was carried out as described above.

### MTT-test and colony formation assay after 5-FU treatment

These methods are provided as Supplementary Methods.

### Isolation of HDAC2-negative subclones

RKO^HDAC2^ cells were harvested and counted manually using a Neubauer chamber. The cell number was adjusted to 10 cells/mL by dilution with DMEM. A total of 100 µL of this solution was applied to each well of multiple 96-well plates. In regular intervals after seeding, the plates were examined under a light microscope, and wells without cells and wells with multiple cell colonies were discarded. Ninety-six wells harboring a single colony of cells were cultured, transferred into 12-well plates, and then seeded in 6-well plates to test for HDAC2 expression via immunoblot and immunofluorescence.

### Protein knockdown with small interfering RNAs

We previously described this method in Göder et al. (2018) and Kiweler et al. (2018).

### Statistical analyses

Statistical analyses were conducted with one-way or two-way ANOVA, as indicated for the respective experiments, using GraphPad Prism Vers.6.01. Correction for multiple testing was achieved with Tukey’s and Sidak’s multiple comparisons test. As measure of significance, *P* values are indicated.

## Results

### Detection and isolation of RKO cells that lack HDAC2

**Fig. 1:**
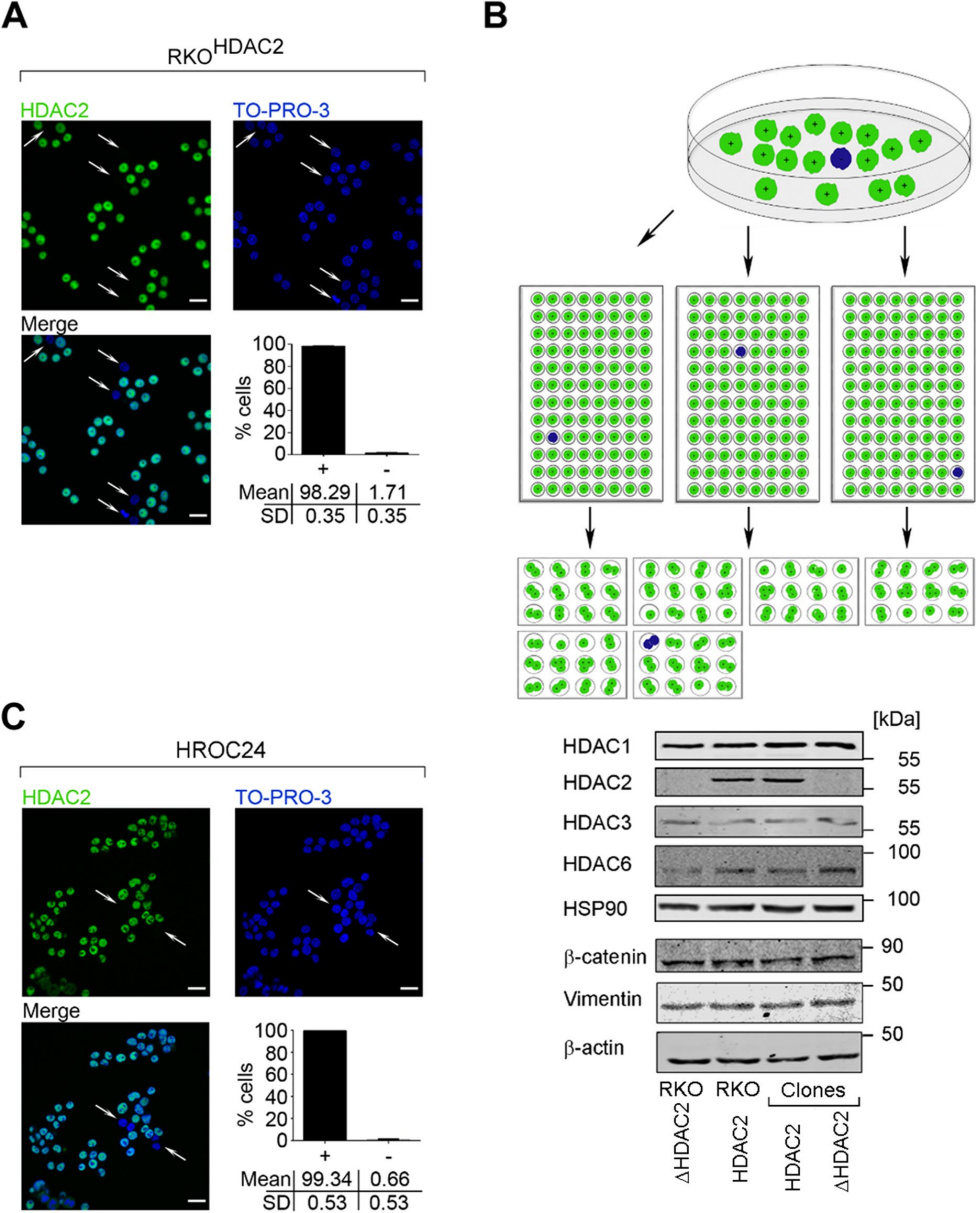
HDAC2 heterogeneity and clonal selection of colorectal cancer cells. **A** Immunofluorescence of untreated RKO^HDAC2^ cells shows heterogeneous HDAC2 expression; representative images; scale bar 20 µm. The quantification represents mean ± SD of RKO cells with HDAC2 ( +) or without HDAC2 ( −) (*n* = 3). B Single-cell isolation of RKO^HDAC2^ cells through limited dilution and immunoblot of resulting HDAC2-positive/negative (RKO^HDAC2^/RKO^ΔHDAC2^) subclones. RKO^ΔHDAC2^ cells (Ropero et al. 2006) were tested in comparison. Green cells represent HDAC2-positive and blue cells HDAC2-negative cells. Lysates of these cells were analyzed by immunoblot as indicated, with HSP90 or β-actin as loading controls. **C** Heterogenous HDAC2 expression in untreated HROC24 cells; representative images; scale bar 20 µm; mean ± SD (*n* = 3)

There is controversy about whether the MSI cell line RKO expresses HDAC2 (Ree et al. 2008; Ropero et al. 2006). Analyzing HDAC2-positive RKO cells (RKO^HDAC2^) by confocal immunofluorescence, we noticed that such cultures contained ~ 1.71 ± 0.35% of cells that lacked HDAC2. These had intact DNA and appeared viable (Fig. 1A). In contrast to these findings for HDAC2, there were no HDAC1-negative cells in RKO cell cultures and HDAC2-positive cells could not be detected in HDAC2-negative RKO cell cultures (RKO^ΔHDAC2^) (Supplementary Fig. S1; these cells were obtained from (Ropero et al. 2006)).

We asked whether cells without HDAC2 were self-perpetuating or merely a bystander population. By limiting dilution, we could repeatedly isolate proliferating HDAC2-negative cell clones from RKO^HDAC2^ cell cultures (Fig. 1B). Like RKO^ΔHDAC2^ cells, these clones lacked HDAC2 expression but retained very similar levels of HDAC1 (Fig. 1B, Supplementary Fig. S2A, S2B). We controlled the immunofluorescence data for HDAC2 by a deliberate mixing of equal amounts of HDAC2-positive and HDAC2-negative RKO cell clones. Immunofluorescence for HDAC2 gave the expected staining pattern (Supplementary Fig. S2C; 50%-positive and 50%-negative for HDAC2).

We then compared the short-tandem-repeat profiles of RKO cells with reference profiles from five cell banks. This fingerprinting verified that all analyzed cell populations were indeed RKO cells - our isolated HDAC2-positive and HDAC2-negative RKO cells obtained by limiting dilution (clones HDAC2/ΔHDAC2), RKO^HDAC2^ cells, and RKO^ΔHDAC2^ cells (Supplementary Table S1 and Fig. S3).

Further analyses illustrated that RKO^HDAC2^ and RKO^ΔHDAC2^ cells expressed very similar levels of HDAC3 and HDAC6. Moreover, these cells had equal levels of β-catenin and vimentin, which are markers of epithelial and mesenchymal cell identity (Fig. 1B). These data disfavor gross differences in the identity of RKO cells with or without HDAC2.

To test whether HDAC2 null cells are present in short-term colorectal cancer cell populations, we stained cultures of HROC24 cells for HDAC2. Further details on these cells can be found in Supplementary Table S2. Such cultures contain ~ 1% of cells without HDAC2 (Fig. 1C). Hence, such cells occur in long-term (Fig. 1A) and short-term (Fig. 1C) colon cancer cell cultures.

These results demonstrate that HDAC2-negative cells are a subpopulation within HDAC2-positive MSI colorectal cancer cell cultures.

### Evaluation of histone acetylation in RKO cells

Due to the unresolved issue of whether RKO cells are sensitive to HDACi (Ree et al. 2008; Ropero et al. 2006), we tested how such cells responded to MS-275. This benzamide blocks the class I HDACs HDAC1, HDAC2, and HDAC3 more selectively (Göder et al. 2018; Kiweler et al. 2018) than the pan-HDACi that were previously used to test the sensitivity of RKO cells to HDACi (Hanigan et al. 2008; Ree et al. 2008; Ropero et al. 2006).

**Fig. 2:**
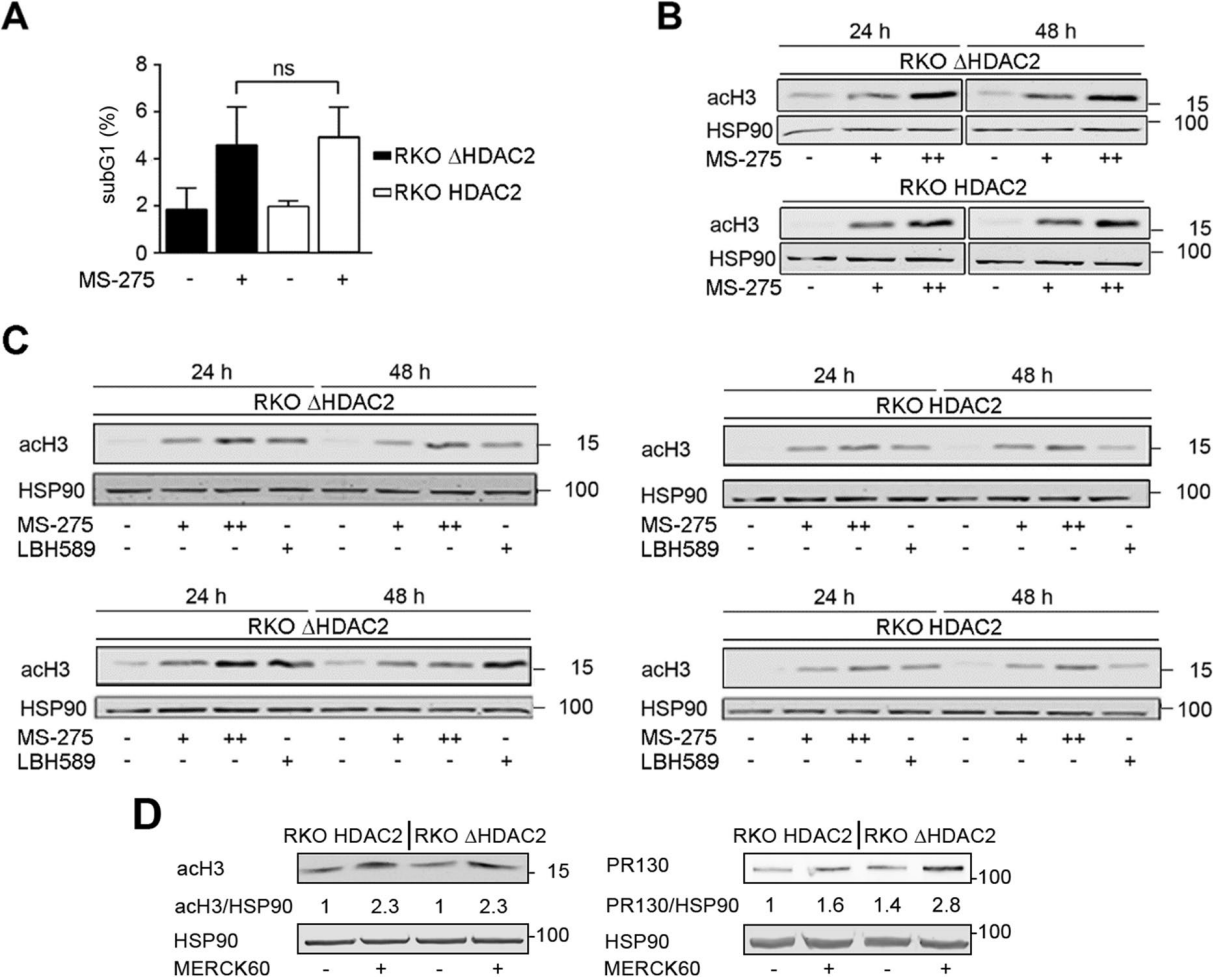
No association between HDAC2 expression and cellular sensitivity to HDACi. **A** RKO^ΔHDAC2^ and RKO^HDAC2^ cells were treated with 5 µM MS-275 for 48 h (+ ; − , cells treated with solvent as control). Cell death was determined as subG1 fractions with fragmented DNA via flow cytometry (mean ± SD; *n* = 4; two-way ANOVA; Sidak multiple comparisons test; ns, not significant). **B** RKO^ΔHDAC2^ and RKO^HDAC2^ cells were treated with 2 µM ( +) or 5 µM (+ +) MS-275 for 24 h and 48 h. Acetylation of histone H3 was analyzed by immunoblot (*n* = 3); HSP90, loading control. **C** Different clones of HDAC2-positive and HDAC2-negative RKO cells (upper row, RKO^ΔHDAC2^ and RKO^ΔHDAC2^ cells; lower row, isolated HDAC2-positive and HDAC2-negative cells that were collected as depicted in Fig. 1B) were treated for 24 h with 2 µM MS-275 ( +), 5 µM (+ +) MS-275, 30 nm LBH589 ( +), or solvent control ( −). Immunoblot was done as indicated. **D** RKO^HDAC2^ and RKO^ΔHDAC2^ cells were treated with 5 µM MERCK60 ( +) for 24 h. Immunoblots were done for acetylated histone H3 (acH3), PR130, and HSP90 (loading control). Values are mean values of two experiments and indicate the band intensities of acH3 and PR130 divided by band intensities of HSP90

We first assessed DNA fragmentation, a marker for cell death due to apoptosis and necrosis, with propidium iodide (PI)-staining and flow cytometry (Marx-Blümel et al. 2017). This experiment showed that RKO^HDAC2^ cells and RKO^ΔHDAC2^ cells were equally responsive to cytotoxic effects of 5 µM MS-275 (Fig. 2A).

Analysis of the acetylation of histones verified (Hanigan et al. 2008; Ree et al. 2008) that HDAC2-positive and HDAC2-negative RKO cells accumulated acetylated histones in response to 2–5 µM MS-275 (Fig. 2B). We could confirm an induction of histone hyperacetylation in four RKO cell types (RKO^HDAC2^, RKO^ΔHDAC2^, and HDAC2-positive/HDAC2-negative clonal cells, shown in Fig. 1B) in response to different concentrations of MS-275 and the hydroxamic acid-based pan-HDACi LBH589 (Fig. 2C).

To analyze histone acetylation in HDAC2-positive and HDAC2-negative RKO cells further, we treated them with MERCK60. This benzamide-based HDACi selectively inhibits HDAC1 and HDAC2 (IC_50_ = 1–8 nM and at least 50-fold selectivity over other HDACs) (Methot et al. 2008). Five micromolar MERCK60 induced an equal accumulation of acetylated histone H3 in both cell types (Fig. 2D). We recently reported that HDAC1 and HDAC2 suppress the transcription of the PP2A subunit PR130 in colorectal cancer cells (Göder et al. 2018). Analysis of PR130 in MERCK60-treated RKO^HDAC2^ and RKO^ΔHDAC2^ cells showed an increase of PR130 in both cell types. This was slightly more evident in RKO cells lacking HDAC2 (Fig. 2D).

These data demonstrate that inhibiting class I HDACs does not affect histone hyperacetylation differentially in RKO cells with or without HDAC2. This agrees with the redundant activities of several HDACs on histones (Müller and Krämer 2010; Witt et al. 2009).

### Responses of RKO cells with various HDAC2 status to 5-FU

Colon tumors are heterogeneous cell populations that respond variably to chemotherapy (Punt et al. 2017). Therefore, we asked whether RKO^HDAC2^ and RKO^ΔHDAC2^ cells responded differently to DNA replication stress and DNA damage. We evoked these conditions with clinically relevant doses of 5-FU (Saif et al. 2009; Wilson et al. 2014). We analyzed cell cycle progression and apoptosis-associated DNA fragmentation by flow cytometry.

**Fig. 3:**
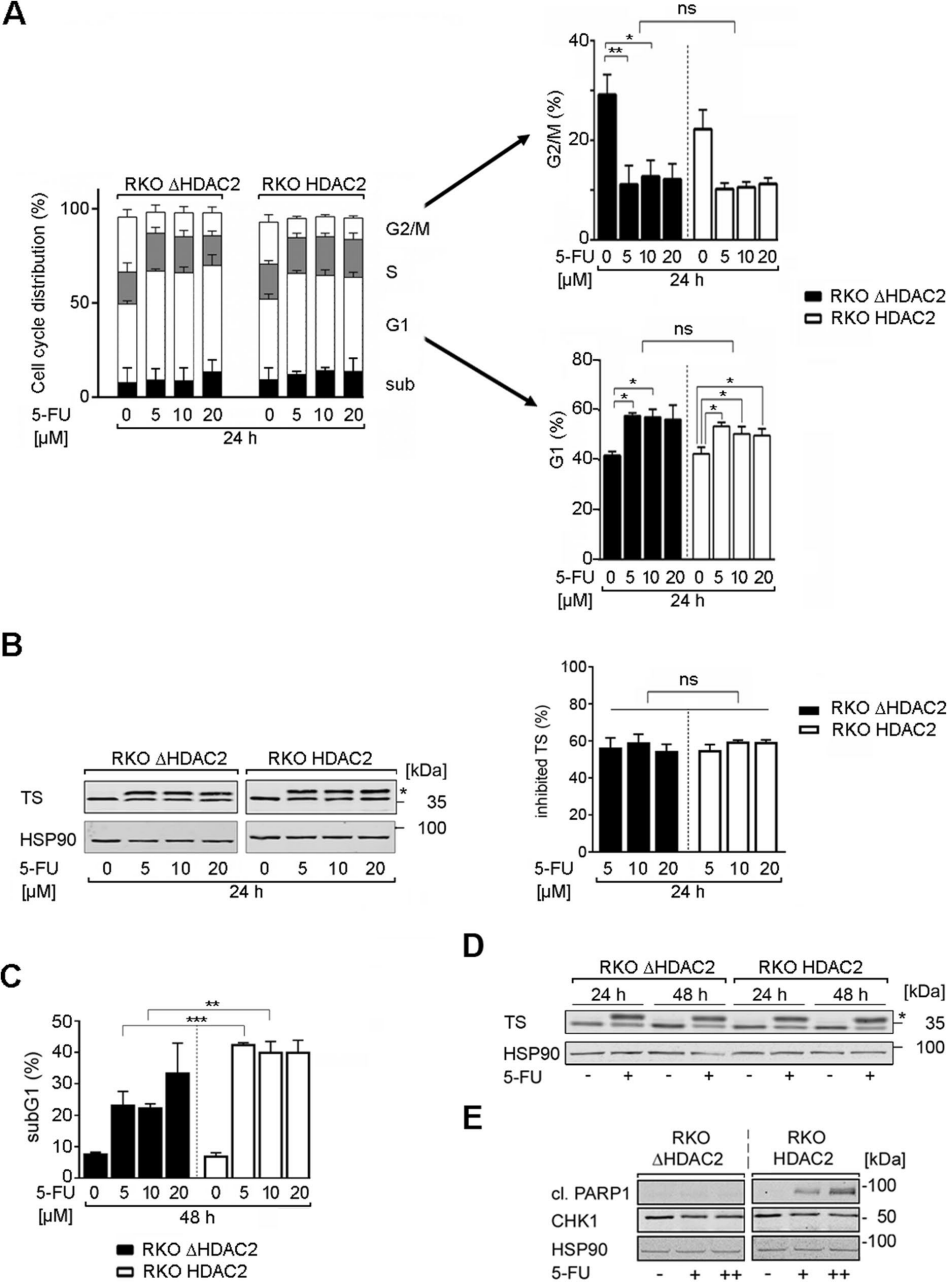
HDAC2 status determines cellular sensitivity to 5-FU. **A** RKO^ΔHDAC2^ and RKO^HDAC2^ cells were treated with 5, 10, and 20 µM 5-FU (24 h). Cell cycle phases were determined by flow cytometry using PI. G1 phases and G2/M phases are altered significantly and are presented separately on the right including statistics (mean ± SD; *n* = 3; one-way ANOVA; Tukey’s multiple comparisons test; *P < 0.05, **P < 0.01). **B** RKO^ΔHDAC2^ and RKO^HDAC2^ cells were treated with 5, 10, and 20 µM 5-FU (24 h), and analyzed for the expression of TS and its slower migrating band by immunoblot (ternary complex of TS, FdUMP, CH_2_THF, marked by an asterisk). The graph shows the percentage of the inhibited TS form (*n* = 3, ns, not significant). **C** RKO^ΔHDAC2^ and RKO^HDAC2^ cells were exposed to 5 µM 5-FU (48 h). The DNA fragmentation that is associated with cell death (cells in subG1 phase) was determined by flow cytometry (mean ± SD; *n* = 3; one-way ANOVA; Tukey’s multiple comparisons test; **P < 0.01, ***P < 0.001). **D** RKO^ΔHDAC2^ and RKO^HDAC2^ cells were treated with 5 µm 5-FU for 24–48 h and analyzed by immunoblot for TS and HSP90 (loading control); *, inhibited TS; *n* = 3. **E** RKO^ΔHDAC2^ and RKO^HDAC2^ cells were exposed to 5 µM 5-FU (24 h). Lysates were processed for immunoblot with antibodies to cleaved PARP1, CHK1, and HSP90 as loading control (*n* = 2)

Treatment with 5 µM 5-FU for 24 h significantly stalled RKO^HDAC2^ and RKO^ΔHDAC2^ cells in the G1 phase of the cell cycle. This was linked to a decrease of cells in the G2/M phase. This reduction of G2/M phase cells was statistically significant in RKO^ΔHDAC2^ cells, but not in RKO^HDAC2^ cells (Fig. 3A). Increasing the dose of 5-FU to 10 µM and 20 µM did not further promote such cell cycle alterations, indicating that 5 µM 5-FU caused a maximal target inhibition. Alterations in the numbers of cells in the S phase and cytotoxic DNA fragmentation were not observed in both RKO cell types after exposure to 5–20 µM 5-FU for 24 h (Fig. 3A).

A primary target of 5-FU is TS (Vodenkova et al. 2020; Wilson et al. 2014; Wyatt and Wilson 2009). 5-FU inhibits TS by forming covalent, ternary complexes consisting of TS, FdUMP, and 5,10-methylene tetrahydrofolate (CH_2_THF). Quantification of free and complexed TS is possible because complexed TS shows a reduced mobility in SDS-PAGE (38 versus 35 kDa). The 38 kDa band is inactive FdUMP-CH_2_THF-TS (Brody et al. 2006). Consistent with the equal impact of 5-FU on the cell cycle progression of RKO^HDAC2^ and RKO^ΔHDAC2^ cells (Fig. 3A), 5-FU caused an equal inhibition of TS in these cells (Fig. 3B). As for the cell cycle alterations (Fig. 3A), increasing the dose of 5-FU from 5 to 10 µM and 20 µM did not further promote TS inhibition (Fig. 3B).

Next, we assessed DNA fragmentation below 2 N (subG1 populations in cell cultures) in response to treatment with 5–20 µM 5-FU for 48 h. RKO^ΔHDAC2^ cells were significantly less sensitive to 5-FU than RKO^HDAC2^ cells (Fig. 3C). Five to 10 µM 5-FU increased cytotoxic DNA fragmentation from 8 up to 23% in RKO^ΔHDAC2^ cells and this increased to 33% with 20 µM 5-FU. Five micromolar 5-FU increased the subG1 fraction from 7 to 42% in HDAC2-positive RKO cells. This was not augmented further with higher doses of 5-FU, indicating a reached plateau of apoptosis induction (Fig. 3C).

As controls for this experiment, we probed immunoblots for the inactivation of TS (Brody et al. 2006) and the apoptosis-associated cleavage of the DNA repair protein PARP1 (Marx-Blümel et al. 2017). Inhibition of TS was equal in both RKO cell types at 24–48 h (Fig. 3D). Immunoblotting for the cleavage product of the caspase-3 target PARP1 verified that 5-FU was more pro-apoptotic for RKO cells with HDAC2. This was not linked to a general protein degradation; CHK1 was expressed about equally in the two cell types (Fig. 3E).

We consequently aimed to collect additional evidence for an HDAC2-dependent sensitivity of cells to 5-FU. Consistent with Fig. 3C, we noted that a transient reduction of HDAC2 by siRNAs in RKO^HDAC2^ cells (Supplementary Fig. S4A) decreased cellular sensitivity to 5-FU. This also held for FdUrd which is a deoxynucleotide of 5-FU that can be incorporated into DNA directly (Supplementary Fig. S4B). Furthermore, HDAC2-negative clones that we isolated from RKO^HDAC2^ cell cultures (Fig. 1B) were less susceptible to apoptosis induction by 5-FU than isolated HDAC2-positive clones. This occurred irrespectively of an equal inhibition of TS by 5-FU (Supplementary Figure S5A, S5B). Moreover, a short-term pulse treatment with 2.5 µM 5-FU decreased the clonogenic growth of RKO^HDAC2^ cells but not of RKO^ΔHDAC2^ cells (Supplementary Figure S6).

From these data, we conclude that HDAC2 expression critically regulates the responses of colorectal cancer cells to 5-FU.

### Selection of HDAC2-negative colorectal cancer cells in response to 5-FU

**Fig. 4:**
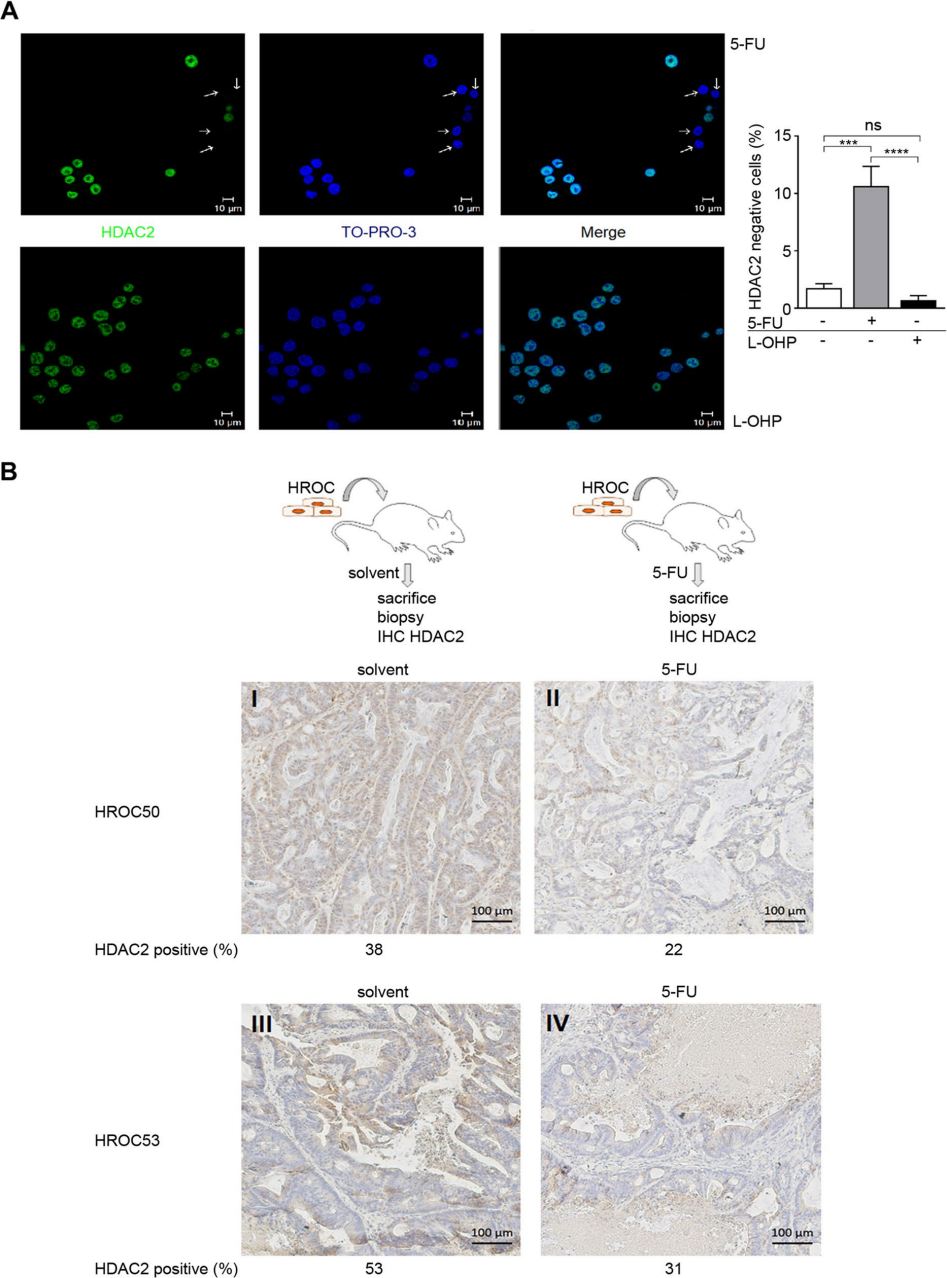
Accumulation of colorectal cancer cells lacking HDAC2 upon long-term treatment with 5-FU. **A** Results from immunofluorescence staining of HDAC2 levels in RKO^HDAC2^ cells following 2 µM 5-FU (upper) or 2 µM L-OHP (lower) for 14 days. Nuclei were stained with TO-PRO-3. Graph on the right shows the numbers of HDAC2-negative RKO cells, mean ± SD, *n* = 3; one-way ANOVA, Tukey post hoc test (***P = 0.001, ****P < 0.001, ns = not significant). B Scheme of the in vivo experiment (upper). Below shown are immunohistochemistry analyses for HDAC2 expression in HROC50 and HROC53 colon cancer tumor biopsies in vivo, after treatment for 18 days with solvent or 5-FU (dose of 20 mg/kg body weight in 100 µl sodium chloride, intraperitoneal, thrice weekly). Treatment with 5-FU led to an accumulation of RKO^ΔHDAC2^ cells

The data above let us hypothesize that prolonged treatment with 5-FU could lead to an accumulation of RKO^ΔHDAC2^ cells. Indeed, treating HDAC2-positive RKO cell cultures with 5-FU for 12 days significantly augmented the percentage of HDAC2-negative cells to ~ 10.6% (Fig. 4A). This effect is not generally induced by chemotherapeutics that disturb DNA replication and integrity. Like 5-FU, the DNA-crosslinking agent L-OHP is frequently used to treat colorectal cancer (Oneda and Zaniboni 2021). Unlike 5-FU, long-term treatment with L-OHP did not evoke an accumulation of HDAC2-negative cells (Fig. 4A).

Differences in proliferation could cause a variable incorporation of 5-FU into nascent DNA in S phase and subsequent DNA damage in RKO^HDAC2^ cells and RKO^ΔHDAC2^ cells. However, the rise in the number of HDAC2-negative cells upon long-term incubation with 5-FU cannot be explained by slower growth kinetics of untreated RKO^ΔHDAC2^ cells. There was a trend that RKO^ΔHDAC2^ cells proliferate slightly faster than RKO^HDAC2^ cells, but this did not reach statistical significance (Supplementary Fig. S7). The outcome of this experiment additionally demonstrates that the differential induction of cell cycle arrest and apoptosis in RKO^HDAC2^ cells and RKO^ΔHDAC2^ cells cannot be explained by different proliferation kinetics. These measurements were done with cells that grew for 24–48 h, which are time periods of very equal growth of these RKO cell isotypes.

To assess a translational relevance of our findings, we transplanted PDX MSI colon cancer tissues (Supplementary Table S2) into immunodeficient mice. Such primary tissues are closer to the patient situation than long-term treated cells (Gao et al. 2015). Established PDX tumors were treated with 5-FU in sodium chloride as a solvent or the solvent alone as a negative control. Mice were sacrificed; tumors were excised, fixed, embedded, and subsequently analyzed by immunohistochemistry for HDAC2 (Fig. 4B). We noted HDAC2-positive and HDAC2-negative colorectal cancer cells in 6 PDX samples (HROC24, HROC29, HROC48, HROC50, HROC53; data not shown). In HROC50 and HROC53 PDX, the treatment with 5-FU led to an accumulation of HDAC2-negative colorectal cancer cells in the tumor mass that remained after treatment (Fig. 4B).

These results illustrate that 5-FU can select for HDAC2-negative cells in a subset of colon tumor cells.

### Evaluation of HDAC2-regulated proteins and sensitivity of colorectal cancer cells to the ATM inhibitor KU-60019

**Fig. 5:**
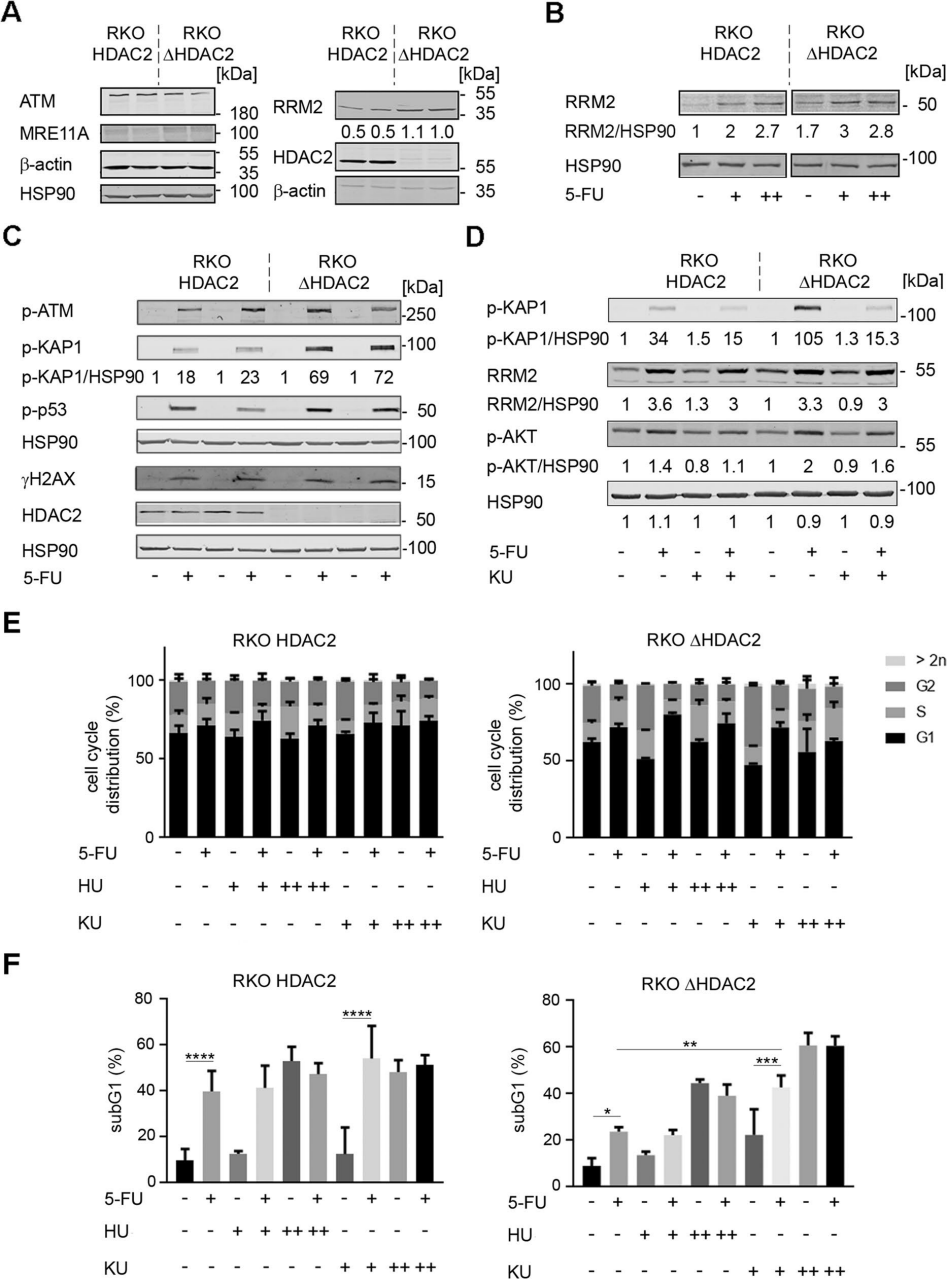
5-FU and KU-60019 favorably combine against RKO cells. **A** RKO^HDAC2^ and RKO^ΔHDAC2^ cells were lysed and immunoblot was carried out as indicated with β-actin and HSP90 as loading controls; *n* = 2. B RKO^HDAC2^ and RKO^ΔHDAC2^ cells were treated with 5 µM ( +) to 10 µM (+ +) 5-FU for 24 h. RRM2 levels were analyzed by immunoblot; HSP90, loading control; *n* = 2. Numbers denote average band intensities of RRM2 divided by HSP90 band intensities; band intensities of RKO^HDAC2^ cells are set as 1. **C** RKO^HDAC2^ and RKO^ΔHDAC2^ cells were incubated with 5 µM (+) 5-FU for 24 h. The indicated proteins/posttranslational modifications of proteins were analyzed by immunoblot; HSP90, loading control; *n* = 4. Numbers denote induction of p-KAP1 in response to 5-FU, measured as band intensities of p-KAP1 divided by band intensities of HSP90; band intensities of untreated cells set as 1. **D** RKO^HDAC2^ and RKO^ΔHDAC2^ cells were incubated with 5 µM (+) 5-FU ± 0.5 µM KU-60019 for 24 h. Immunoblot was done as indicated; *n* = 2. Numbers denote induction of p-KAP1, RRM2, p-AKT in response to 5-FU ± KU-60019, measured as band intensities divided by band intensities of HSP90; band intensities of untreated cells set as 1. **E** RKO^HDAC2^ and RKO^ΔHDAC2^ cells were treated with 5 µM 5-FU (+), 0.1–0.5 mM (+ / + +) hydroxyurea (HU), 0.5–3 µM KU-60019 (+ / + +) for 48 h, and cell cycle profiles were monitored by flow cytometry; mean ± SD; *n* = 3. **F** The same cell populations were analyzed for subG1 fractions by flow cytometry; mean ± SD; *n* = 3.

We investigated by immunoblot whether the differential sensitivity of RKO^HDAC2^ and RKO^ΔHDAC2^ cells towards 5-FU was linked to variable expression levels of proteins that modulate the cellular susceptibility to this drug (Vodenkova et al. 2020). We probed for ATM, MRE11A, and the RNR subunit M2 (RRM2). ATM was equally detectable in RKO^HDAC2^ and RKO^ΔHDAC2^ cells. Consistent with the literature (Giannini et al. 2004; Miquel et al. 2007), we could not detect MRE11A in MSI RKO cells. RRM2 was reproducibly expressed at up to twofold higher levels in RKO^ΔHDAC2^ cells (Fig. 5A). Yet, 5-FU induced RRM2 to about equal levels in 5-FU-treated RKO^HDAC2^ and RKO^ΔHDAC2^ cells (Fig. 5B).

Next, we compared the phosphorylation-dependent activation of ATM in 5-FU-treated RKO^HDAC2^ and RKO^ΔHDAC2^ cells. Phosphorylation of ATM was very similar in both cell types (Fig. 5C). However, the phosphorylation of the ATM target KRAB-associated protein 1 (KAP1; also known as E3 SUMO/ubiquitin protein ligase tripartite motif-containing-28/transcriptional intermediary factor-1β) (Cheng et al. 2014; Hu et al. 2012) was different in the two genotypes. 5-FU induced the phosphorylation of KAP1 at serine-824 about threefold more potently in RKO^ΔHDAC2^ cells than in RKO^HDAC2^ cells (Fig. 5C, D).

The tumor suppressor p53 and the DNA stress sensor histone H2AX are phosphorylated by ATM and further checkpoint kinases (Collins et al. 2020; Kopp et al. 2019; Terabayashi and Hanada 2018). Serine-15 phosphorylated p53 and serine-139 phosphorylated H2AX (ɣH2AX) accumulated about equally in HDAC2-positive and HDAC2-negative cells (Fig. 5C and Supplementary Fig. S5B).

Since KAP1 can prevent apoptosis induction upon DNA damage (Cheng et al. 2014; Hu et al. 2012), we speculated that a suppression of p-KAP1 by the specific ATM inhibitor KU-60019 (Golding et al. 2009; Nguyen et al. 2021) would sensitize HDAC2-negative RKO cells to 5-FU. KU-60019 suppressed the ATM-catalyzed phosphorylation of KAP1 in response to 5-FU in RKO^HDAC2^ cells and RKO^ΔHDAC2^ cells. Unlike this strong impact of KU-60019 on p-KAP1, the induction of RRM2 by 5-FU was weakly affected by KU-60019 (Fig. 5D).

KU-60019 was reported to block pro-survival signaling through ATM-dependent phosphorylation of the AKT kinase at serine-473 (Golding et al. 2009). Consistent herewith, KU-60019 attenuated the basal and 5-FU induced phosphorylation of AKT. Unlike KAP1, AKT was modulated weakly by 5-FU and similarly in RKO^HDAC2^ cells and RKO^ΔHDAC2^ cells (Fig. 5D).

These results encouraged us to test whether KU-60019 sensitized HDAC2-negative RKO cells to 5-FU. Due to the differences in basal RRM2 levels (Fig. 5A), we additionally inhibited RRM2 with its specific inhibitor hydroxyurea (Göder et al. 2018). We carried out flow cytometry assessing the cell cycle profiles of RKO cells (G1-S-G2/M phase cells, exclusion of subG1 fraction). In agreement with Fig. 3A, this assay confirmed that 5-FU increased the amounts of RKO^ΔHDAC2^ and RKO^HDAC2^ cells in G1 phase and reduced G2/M phase cells, with stronger effects in RKO^ΔHDAC2^ cells (Fig. 5E). A total of 0.5 mM hydroxyurea increased the G2/M phase populations in RKO^ΔHDAC2^ cell cultures and slightly altered the cell cycle of RKO^HDAC2^ cells. Three millimolar hydroxyurea increased the S phase populations in RKO^HDAC2^ and RKO^ΔHDAC2^ cells. These data are consistent with a dose-dependent inhibition of RNR by hydroxyurea. A total of 0.5 µM KU-60019 increased the number of RKO^ΔHDAC2^ cells in G2/M phase and this effect was lost when 3 µM KU-60019 were applied. Combinations of hydroxyurea and 5-FU increased the numbers of cells in G1 phase. Moreover, 5-FU prevented the accumulation of RKO^ΔHDAC2^ cells in G2/M phase by KU-60019. The impact of hydroxyurea and KU-60019 on the cell cycle progression of RKO^HDAC2^ cells was less pronounced (Fig. 5E).

Analysis of apoptosis induction verified (Figs. 3C, 3E) that RKO^HDAC2^ cells were significantly more sensitive to 5-FU than RKO^ΔHDAC2^ cells (Fig. 5F; *P < 0.5 versus ****P < 0.001). Three millimolar hydroxyurea induced apoptosis in both RKO cell types, with a trend for a higher sensitivity of RKO^HDAC2^ cells. 5-FU and hydroxyurea did not combine favorably against RKO^ΔHDAC2^ and RKO^HDAC2^ cells. Although 0.5 µM KU-60019 did not significantly increase the subG1 fraction in RKO^ΔHDAC2^ and RKO^HDAC2^ cells, it significantly sensitized RKO^ΔHDAC2^ to 5-FU (*P < 0.5 versus ***P < 0.001). The combined application of 5 µM 5-FU and 0.5 µM KU-60019 triggered 42.8% apoptosis in RKO^ΔHDAC2^ cell cultures (Fig. 5F). Coherent herewith, a 24-h pulse treatment with 5-FU and KU-60019 could attenuate the clonogenic growth potential of RKO^ΔHDAC2^ cells and only allowed the formation of much smaller colonies than in untreated conditions (Supplementary Figure S8). In RKO^HDAC2^ cells, 0.5 µM KU-60019 increased apoptosis-associated DNA fragmentation from 39.8 to 56.2% (Fig. 5F). Measurement of cell vitality with the MTT-test corroborated that 5-FU plus KU-60019 effectively stalled the growth of both RKO^HDAC2^ cells and RKO^ΔHDAC2^ cells (Supplementary Figure S9). A higher dose of 3 µM KU-60019 showed high single-agent activity. It induced 60.7% apoptosis in RKO^ΔHDAC2^ cells and 48.3% apoptosis in RKO^HDAC2^ cells. These numbers were not increased by co-administration of 5-FU (Fig. 5F).

These data show that ATM is a valid target in RKO cells and that ATM inhibition can sensitize RKO cells to 5-FU.

## Discussion

Coding microsatellite mutations in the *HDAC2* gene locus were detected in up to 43% of MSI primary tumors (Hanigan et al. 2008). In its first exon, the *HDAC2* gene contains 9 adenine repeats which are frequently mutated in MSI. A homozygous 1-bp deletion was detected in RKO cells and heterozygous + 1/ − 1 mutations can occur in long-term passaged HCT116 cells and SW48 cells (Hanigan et al. 2008; Ropero et al. 2006). Hence, even cells without MSI can contain HDAC2-negative subpopulations. In light of the high level of colorectal cancer intra-tumoral heterogeneity (Punt et al. 2017), one can assume that HDAC2 is frequently mutated in sub-clonal tumor cell populations. It is possible that certain culture conditions favor an outgrowth of HDAC2-negative cells. For example, endogenous DNA replication stress due to oncogene activation (Dobbelstein and Sørensen 2015) may favor the outgrowth of such cells. This would correspond to a common scheme in evolutionary biology, the Darwinian selection of the fittest subpopulations upon changes in the environment (Bell and Gilan 2020).

Our data demonstrate that HDAC2-negative RKO cell cultures become less apoptotic than HDAC2-positive RKO cell cultures when they are treated with 5-FU, which is a strong inducer of cell stress. While this suggests that HDAC2 is a novel molecular marker and gatekeeper for apoptosis resistance to 5-FU, additional data are needed to test whether a lack of HDAC2 in significant parts of tumor mass can explain why subsets of MSI are not successfully treated with 5-FU (Vilar and Gruber 2010). Moreover, pro-apoptotic or growth-arresting properties of 5-FU are different in HDAC2-positive and HDAC2-negative RKO cells. Depending on the tumor type, both processes can be crucial for the treatment of individual tumors. Independent of such details, a remaining tumor mass of 10% or far less can be relevant. Tumors consist of billion cells which can spread and give rise to deadly metastases. Thus, it is a prime goal to eliminate all cancer cells hard and early.

The similar growth kinetics of RKO cells with or without HDAC2 suggest that there is no proliferative pressure on randomly occurring HDAC2-negative populations. Nevertheless, we noted a trend for a faster proliferation of HDAC2-negative cells in vitro. This result, which is consistent with the reported role of HDAC2 in the regulation of cell cycle progression (Yamaguchi et al. 2010; Zhu et al. 2004), may be a result of the higher expression of RRM2 in HDAC2-negative RKO cells. RRM2 is the catalytic subunit of RNR which provides the building blocks for DNA synthesis (Neitzel et al. 2020; Peters et al. 2002; Vodenkova et al. 2020). Higher levels of RRM2 in HDAC2-negative cells may also be the reason why we did not see HDAC2-positive cells in HDAC2-negative RKO cell cultures. Irrespective thereof, the presence of HDAC2 in most cancer cells suggests that its expression is more beneficial than its loss in most tumor cells. That RKO^ΔHDAC2^ cell populations have a reproducible trend for higher levels of G2/M phase cells than RKO^HDAC2^ cell populations may indicate that G2/M phase defects disfavor a more generalized loss of HDAC2.

Independent of the link between HDAC2 and RRM2, this study shows that inhibition of RRM2 with HU does not combine favorably with 5-FU against RKO cells. Such a finding can be explained by two activities of RRM2 and their antagonistic effects on toxicity induced by 5-FU. On the one hand, higher levels of RRM2 can increase the intracellular concentrations of dNTPs that can outcompete the incorporation of the DNA-damaging 5-dFUTP by replicative DNA-polymerases. On the other hand, higher levels of RRM2 can lead to enhanced synthesis of FdUDP, which can be metabolized and incorporated as FdUTP into DNA by replicative DNA-polymerases (Vodenkova et al. 2020). Dissimilar for RRM2, HDAC2 had no notable impact on the expression of TS and 5-FU inhibited TS irrespective of HDAC2. This illustrates that this very early mechanism of 5-FU is independent of HDAC2.

The MRN complex (MRE11-RAD50-NBS1) activates the phosphorylation of ATM (Paull 2015; Terabayashi and Hanada 2018). Our data show that RKO cells activate ATM phosphorylation without having detectable levels of MRE11 (Giannini et al. 2004; Miquel et al. 2007). Since ATM can be phosphorylated independently of MRN by ATR in response to replication stress induction by HU and UV-light (Stiff et al. 2006), we assume that 5-FU triggers direct phosphorylation of ATM by ATR. Such an activation of ATR, which implies activation of the ATR/ATM downstream targets CHK1/CHK2, can well explain the equal phosphorylation of the checkpoint kinase substrates p53 and H2AX in HDAC2-positive and HDAC2-negative RKO cells that are exposed to 5-FU.

Contrasting the similar activation of ATM, 5-FU induces phosphorylation of the ATM target p-KAP1 more strongly in HDAC2-negative than in HDAC2-positive RKO cells. Our experiments with KU-60019 suggest that this may protect HDAC2-negative RKO cells from 5-FU. The lower sensitivity of such cells to 5-FU might be explained by an anti-apoptotic function of p-KAP1 during DNA damage (Cheng et al. 2014; Hu et al. 2012). Since KAP1 can be subjected to reversible lysine acetylation (Lin et al. 2015), we tested whether HDAC2 determines KAP1 acetylation. Immunoprecipitation of p-KAP1 followed by immunoblot for acetylation demonstrated an equal acetylation of p-KAP1 in RKO^HDAC2^ cells and RKO^ΔHDAC2^ cells (Nguyen and Krämer, unpublished results). This lack of impact of HDAC2 is consistent with the control of KAP1 acetylation by another deacetylase, which is SIRT1, and the finding that deacetylation of KAP1 does not crosstalk with the DNA damage-induced phosphorylation of KAP1 (Lin et al. 2015). Due to hundreds of ATM substrates (Matsuoka et al. 2007), future studies are needed to exactly define the ATM-dependent phospho-proteome that determines the cytotoxicity of 5-FU. These proteins can also contribute to ATM-dependent mechanisms that promote DNA repair by homologous recombination (Terabayashi and Hanada 2018), which is controversially discussed to be activated or repressed by 5-FU (Huehls et al. 2016; Ito et al. 2020; Srinivas et al. 2015; Wilson et al. 2014; Wyatt and Wilson 2009). It is tempting to speculate that the inhibition of ATM hampers the removal of incorporated, 5-FU-derived nucleotides and additionally compromises homologous recombination. Curiously, pharmacological inhibition of ATR sensitized cancer cells to 5-FU, too, but this occurred independent of homologous recombination (Ito et al. 2020).

Like colorectal cancer cells, endometrial and gastric cancers show frequent MSI and contain subpopulations of cells that lack HDAC2 (Ropero et al. 2006). Studies are underway to define whether such cells respond favorably to combinations of 5-FU and inhibitors of ATM.

## Conclusions

Heterogeneity of tumor cell populations poses a challenge for clinical treatment schemes. Our data suggest that a pharmacological inhibition of ATM might be a novel therapeutic option for tumor cells surviving 5-FU. Experiments in a larger panel of cell lines and in mouse models are required to evaluate the clinical utility of such combinatorial treatments.

### Supplementary Information

Below is the link to the electronic supplementary material.

Supplementary file1 (DOCX 1517 KB)

Supplementary file2 (PPTX 39039 KB)

Supplementary file3 (DOCX 24 KB)

Supplementary file4 (DOCX 140 KB)

## Data Availability

All data generated or analyzed during this study are included in this published article (and its supplementary information files). Uncropped images of all immunoblot data, including the molecular weight marker bands, are included as supplementary material.

## Abbreviations

ATM: Ataxia-telangiectasia mutated
5-FU: 5-Fluorouracil
HDAC: Histone deacetylase
HDACi: Histone deacetylase inhibitor
HU: Hydroxyurea
KAP1: KRAB-associated protein-1/tripartite motif-containing-28/transcriptional intermediary factor-1β
L-OHP: Oxaliplatin
RRM2: Ribonucleotide reductase subunit M2
MSI: Microsatellite instability
PI: Propidium iodine
TS: Thymidylate synthase
