# Supplementary material for: The epigenetic modifier HDAC2 and the checkpoint kinase ATM determine the responses of microsatellite instable colorectal cancer cells to 5-fluorouracil
Source: Cell Biol Toxicol. 2022 May 24;39(5):2401–19. doi: 10.1007/s10565-022-09731-3 (PMC10547618; doi:10.1007/s10565-022-09731-3)
Supplement: Supplementary file 1 — Supplementary file1 (DOCX 1517 KB) [file 10565_2022_9731_MOESM1_ESM.docx]

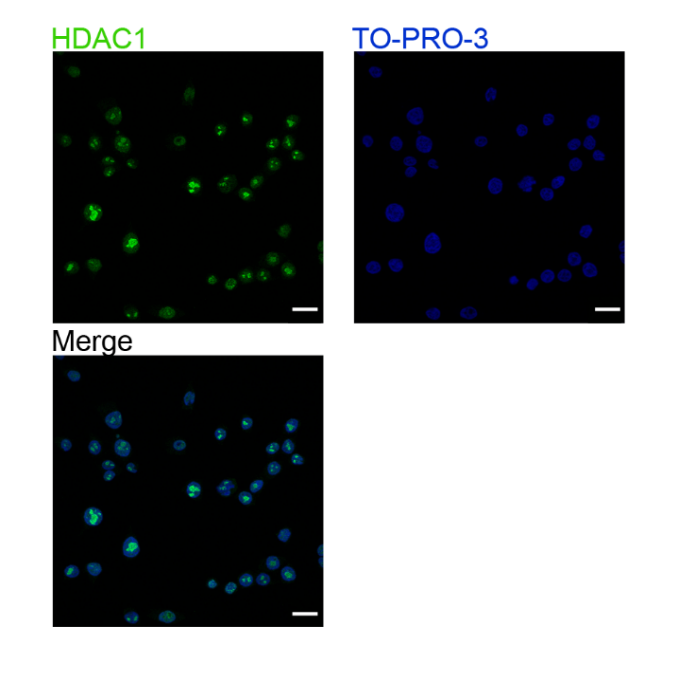


**Supplementary Figure S1. Immunofluorescence analysis for HDAC1 expression in RKO^HDAC2^ cells.** Representative images are shown. Scale bar corresponds to 20 µm.


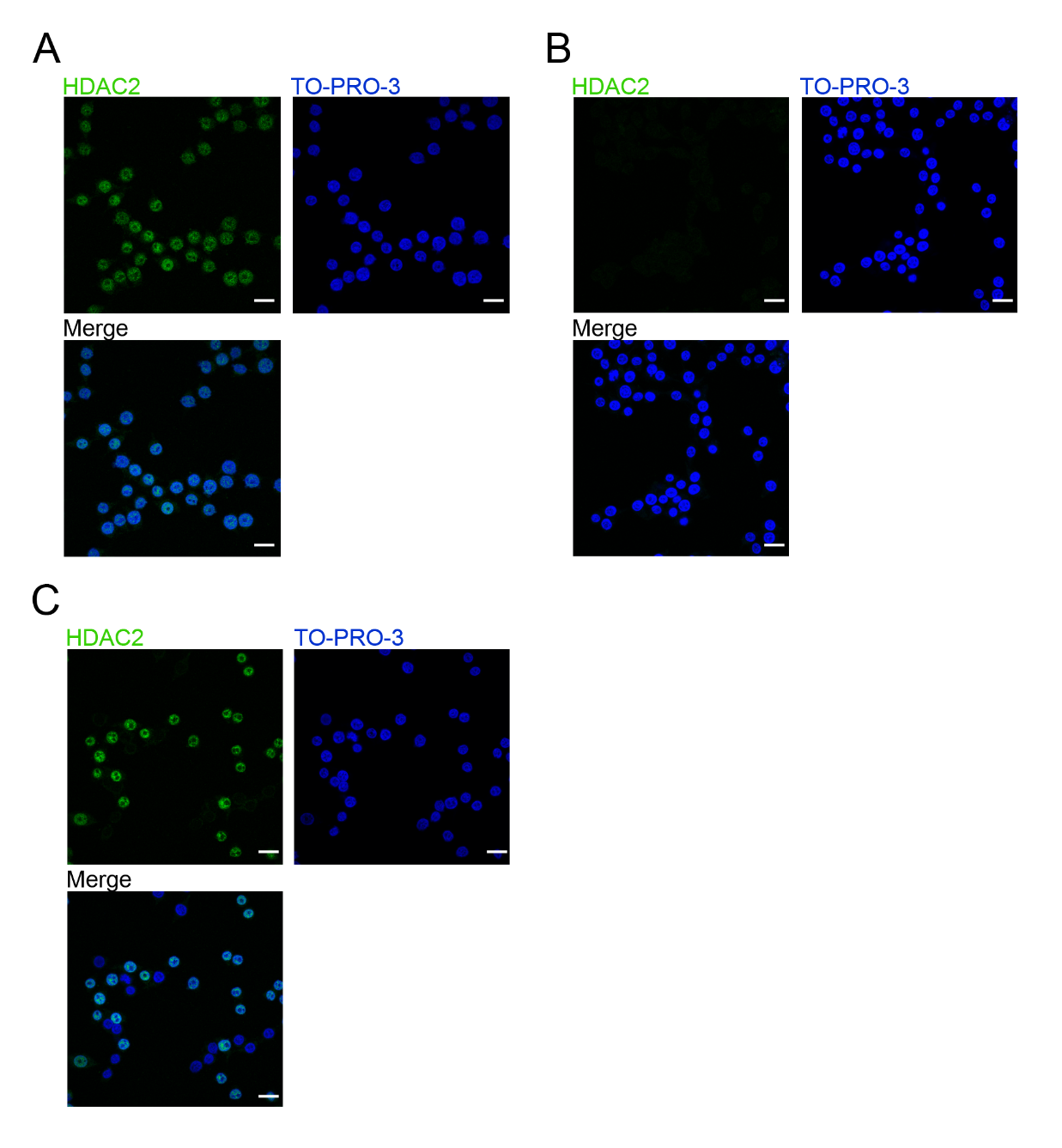


**Supplementary Figure S2. Immunofluorescence analysis with HDAC2 antibodies was done with HDAC2-positive and HDAC2-negative subclones.** Representative images are shown. Scale bar corresponds to 20 µm. (**A**) RKO HDAC2-positive subclone; (**B**) RKO HDAC2-negative subclone; (**C**) RKO HDAC2-positive and HDAC2-negative subclones were seeded in a ratio of 1:1. The obtained data verify the expected 1:1 ratio of HDAC2-positive to HDAC2-negative cells. See main text for details on immunofluorescence analysis.

**Supplementary Figure S3. STR electrograms for DNA fingerprinting analysis.** Respective STR electrograms of DNA fingerprinting analysis of RKO^ΔHDAC2^ cells (RKO ES.A04_170801068D), RKO^HDAC2^ cells (RKO.H03_170801068BC), HDAC2-positive RKO cell clones (RKO HDAC2 pos.B04_170801068F), and HDAC2-negative RKO cell clones (RKO HDAC2 neg.C04_17080106BG) that we isolated from RKO^HDAC2^ cells (see main text, **Fig. 1B**, for details).

**
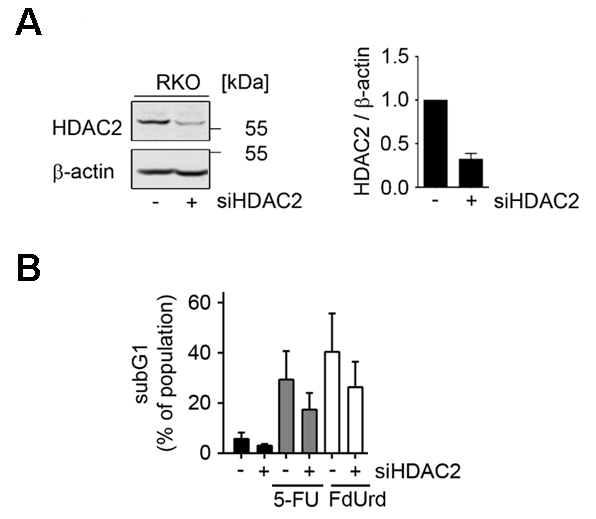
**

**Supplementary Figure S4. Attenuation of HDAC2 by siRNAs decreases sensitivity of RKO cells to 5-FU.** (**A**) HDAC2-positive RKO cells were transfected with siRNA directed against HDAC2 (+; -, irrelevant control siRNA). Reduction of protein expression after 96 h was quantified with immunoblot as fluorescence signal intensity normalized to β-actin loading control. Graph shows mean ± SD of two independent analyses. (**B**) RKO cells that were transfected as in (A) were treated with 5 μM 5-FU or 2 μM FdUrd for 48 h (+, treated; -, solvent-treated). Cell death was analyzed with flow cytometry using PI-staining and subG1 analysis. Graph shows mean ± SD of three independent experiments.


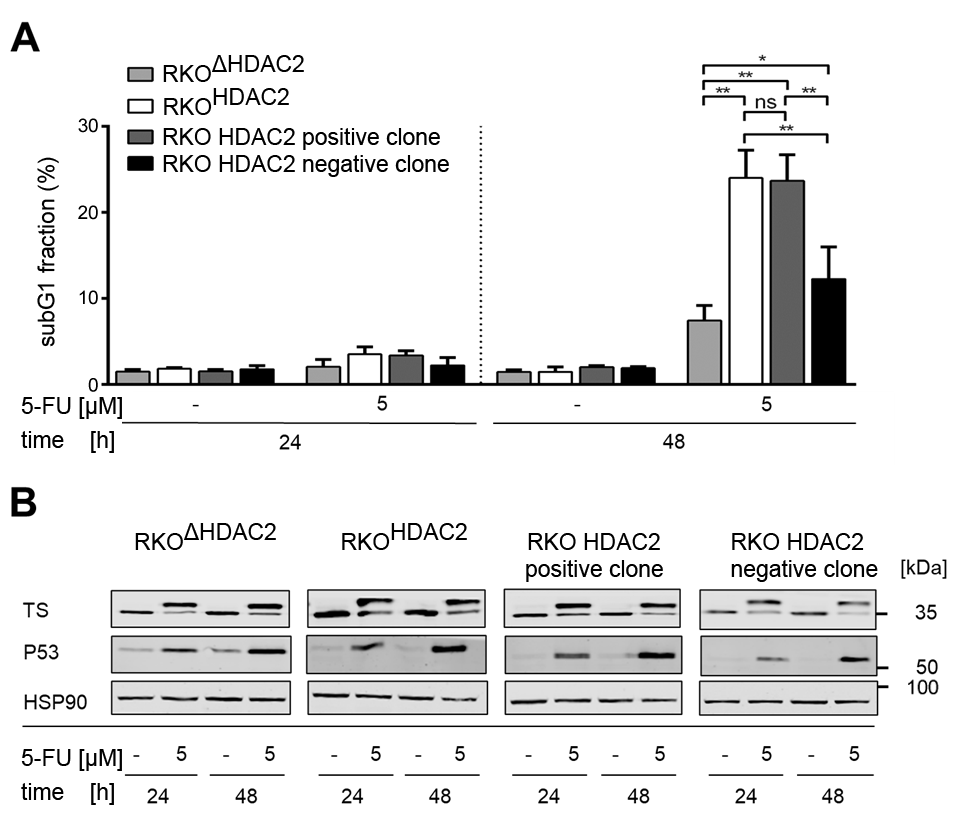


Supplementary Figure S5. Cell cycle analysis and immunoblot after treatment with 5 µM 5-FU. (A) Shown are the mean +/- SD values of the percentages of cells in subG1. RKO^ΔHDAC2^, RKO^HDAC2^, and RKO HDAC2-positive and RKO HDAC2-negative cell clones (see main text, Fig. 1B, for details) were treated with 5 µM 5-FU for 24-48 h or were left untreated (-). Data are from three independent experiments. Variance was calculated using Tukey's one-factorial ANOVA and post hoc test; * = p<0.05; ** = p<0.0001; ns = not significant. (B) Immunoblot analyses was used to analyze the expression of TS and its inhibited form (upper band), p53, and HSP90 in the four different RKO cell lines. Shown are data for the two endpoints after treatment with 5 µM 5-FU for 24 h and 48 h and untreated controls (-).


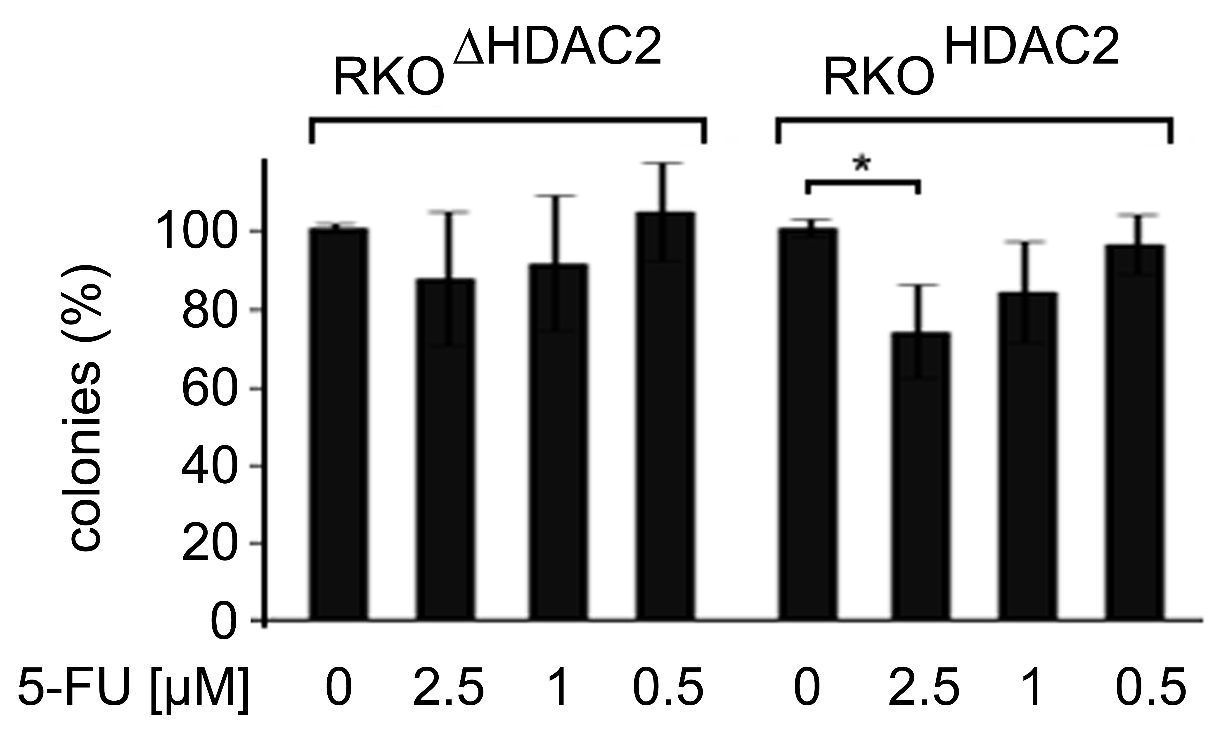


Supplementary Figure S6. Colony formation of RKO cells after pulse treatment with 5-FU. RKO^ΔHDAC2^ cells and RKO^HDAC2^ cells (500 cells/dish) were incubated with the indicated concentrations of 5-FU for 24 h. 10 d later, colony formation was assessed; n=2, p<0.05, t-test.

**
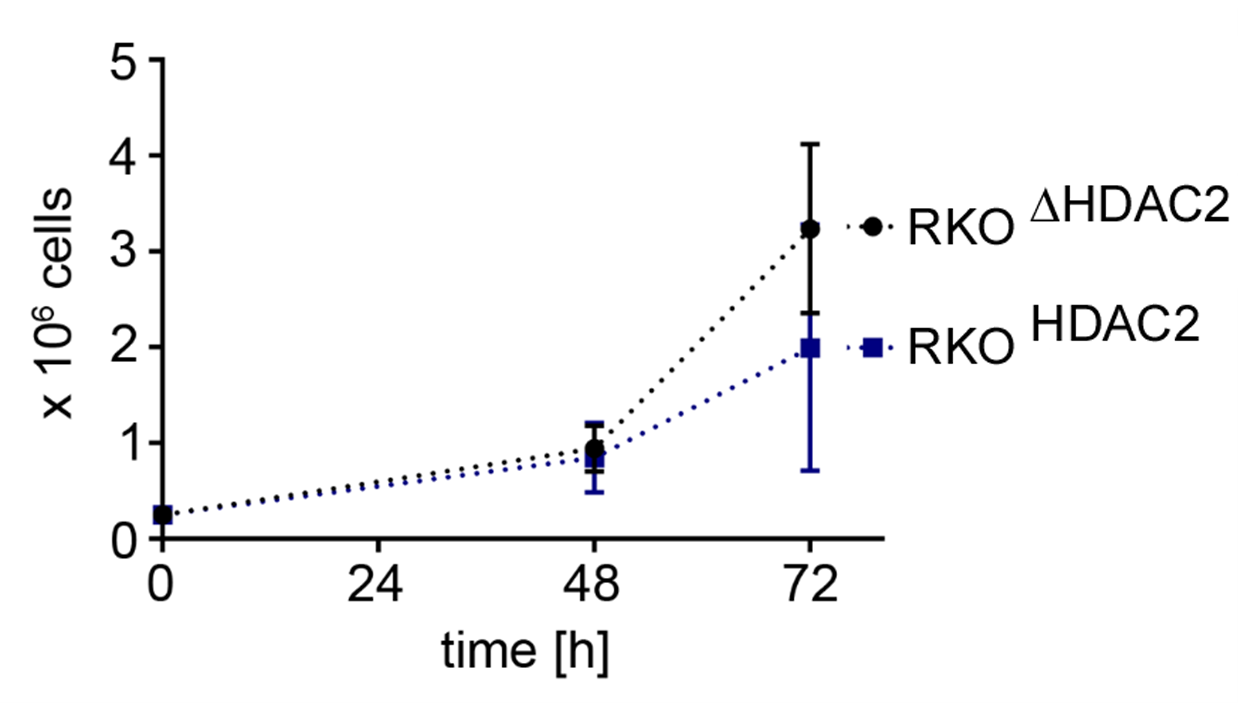
**

**Supplementary Figure S7. Proliferation of** **RKO^ΔHDAC2^ and RKO^HDAC2^ cells.** 0.25 x 10^6^ RKO^ΔHDAC2^ cells and RKO^HDAC2^ cells cells were seeded and their growth after 48 and 72 h was determined by cell counting (means ± SD; n = 3-4).


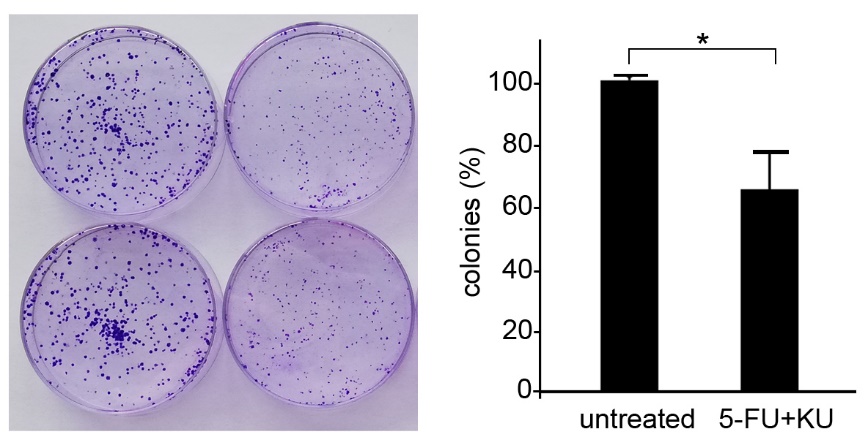


Supplementary Figure S8. Pulse treatment with 5-FU and KU-60019 suppresses chlorogenic growth of HDAC2-negative RKO cells. 500 HDAC2-negative RKO cells (RKO^ΔHDAC2^) were seeded in 6 cm plates, incubated with 2.5 µM 5-FU and 1.5 µM KU-60019 for 24 h, washed, and analyzed for cell growth 10 d later (n=2, p<0.05, t-test).

.


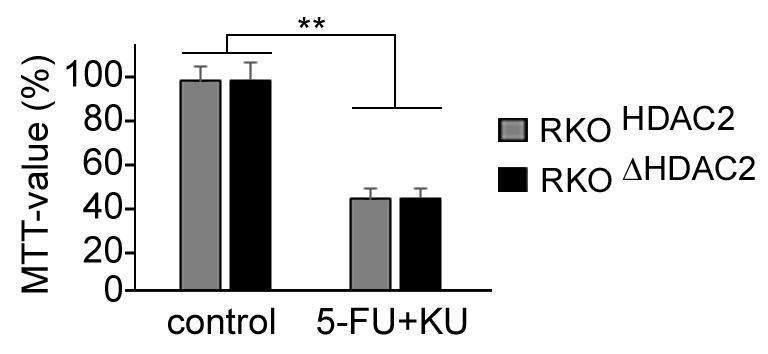


Supplementary Figure S9. RKO cells are susceptible to growth arrest induction by 5-FU and KU-60019. 10^4^ HDAC2-negative and HDAC2-positive RKO cells were seeded in 96-well plates, incubated with 5 µM 5-FU ± 3 µM KU-60019 for 48 h, and analyzed by MTT-test (n=2, p<0.01, t-test).
