# Supplementary material for: The epigenetic modifier HDAC2 and the checkpoint kinase ATM determine the responses of microsatellite instable colorectal cancer cells to 5-fluorouracil
Source: Cell Biol Toxicol. 2022 May 24;39(5):2401–19. doi: 10.1007/s10565-022-09731-3 (PMC10547618; doi:10.1007/s10565-022-09731-3)
Supplement: Supplementary file 2 — Supplementary file2 (PPTX 39039 KB) [file 10565_2022_9731_MOESM2_ESM.pptx]

## Slide 1
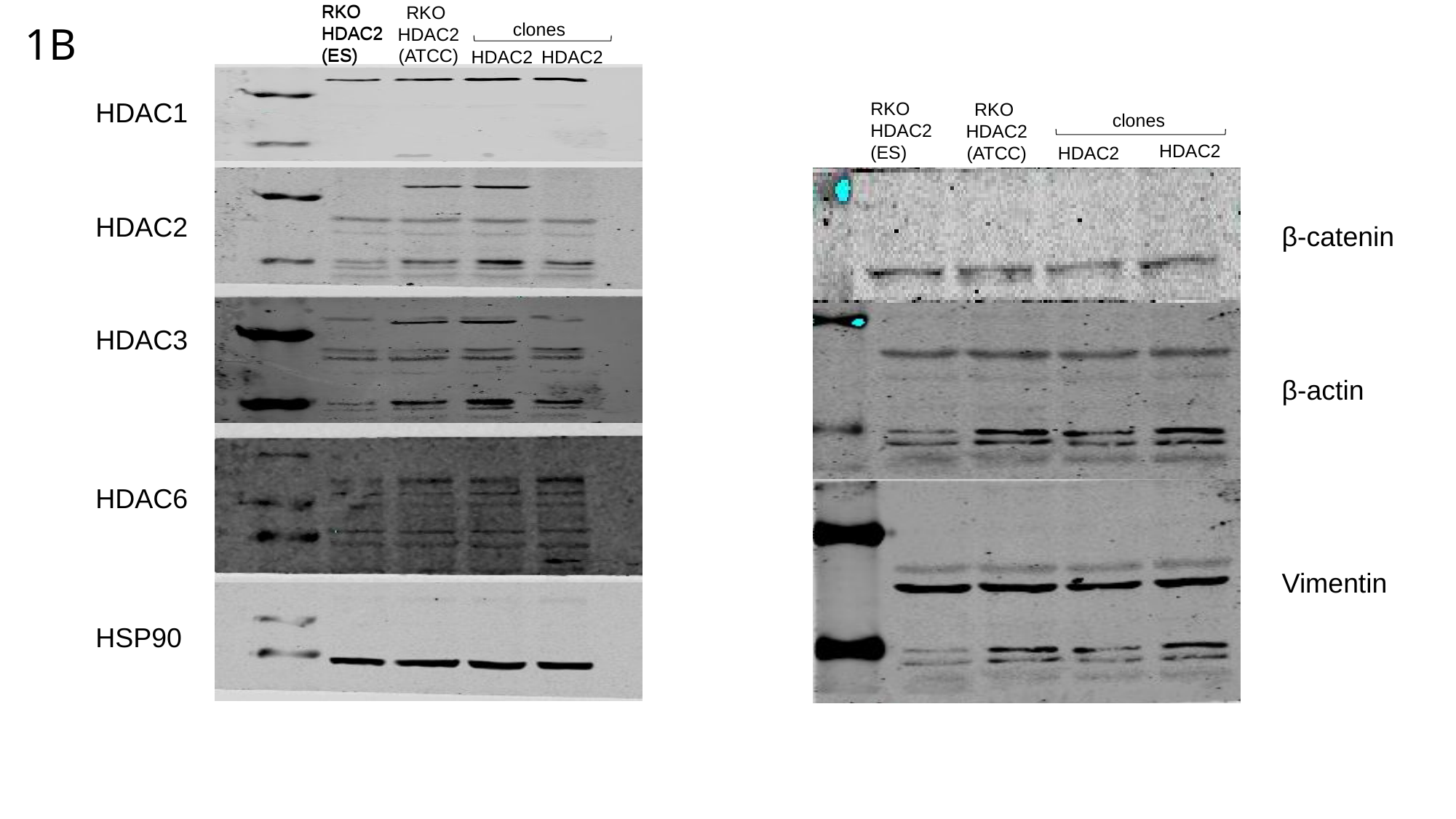

RKO
HDAC2
(ATCC)
1B
clones
HDAC2
HDAC1
RKO
HDAC2
(ATCC)
clones
HDAC2
HDAC2
β-catenin
HDAC3
β-actin
HDAC6
Vimentin
HSP90

## Slide 2
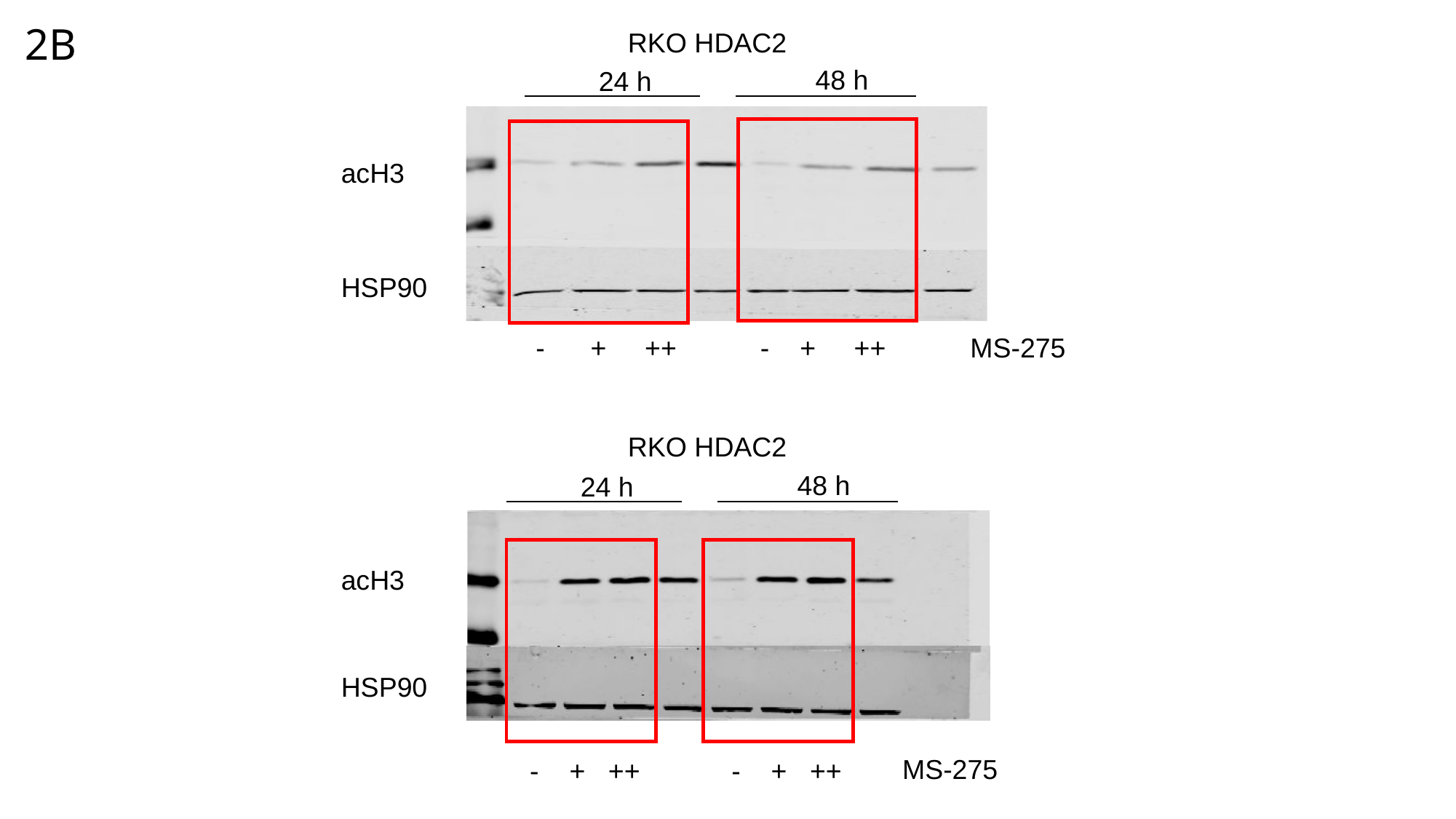

2B
48 h
24 h
acH3
HSP90
 - + ++ - + ++
MS-275
RKO HDAC2
48 h
24 h
acH3
HSP90
MS-275
 - + ++ - + ++

## Slide 3
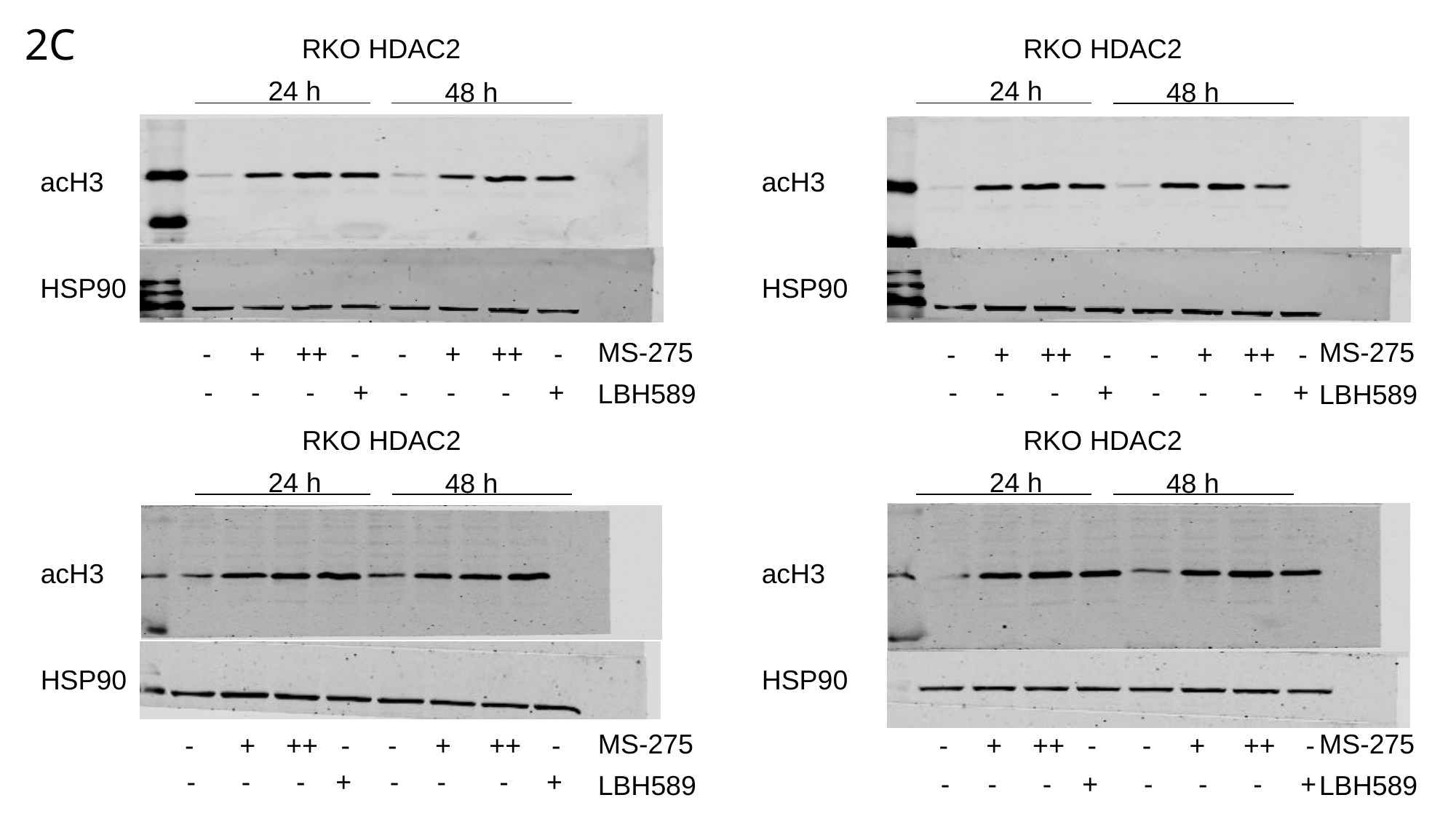

2C
RKO HDAC2
24 h
24 h
48 h
48 h
acH3
acH3
HSP90
HSP90
MS-275
MS-275
- + ++ - - + ++ -
 - + ++ - - + ++ -
- - - + - - - +
 - - - + - - - +
LBH589
LBH589
RKO HDAC2
24 h
24 h
48 h
48 h
acH3
acH3
HSP90
HSP90
MS-275
MS-275
- + ++ - - + ++ -
 - + ++ - - + ++ -
- - - + - - - +
 - - - + - - - +
LBH589
LBH589

## Slide 4
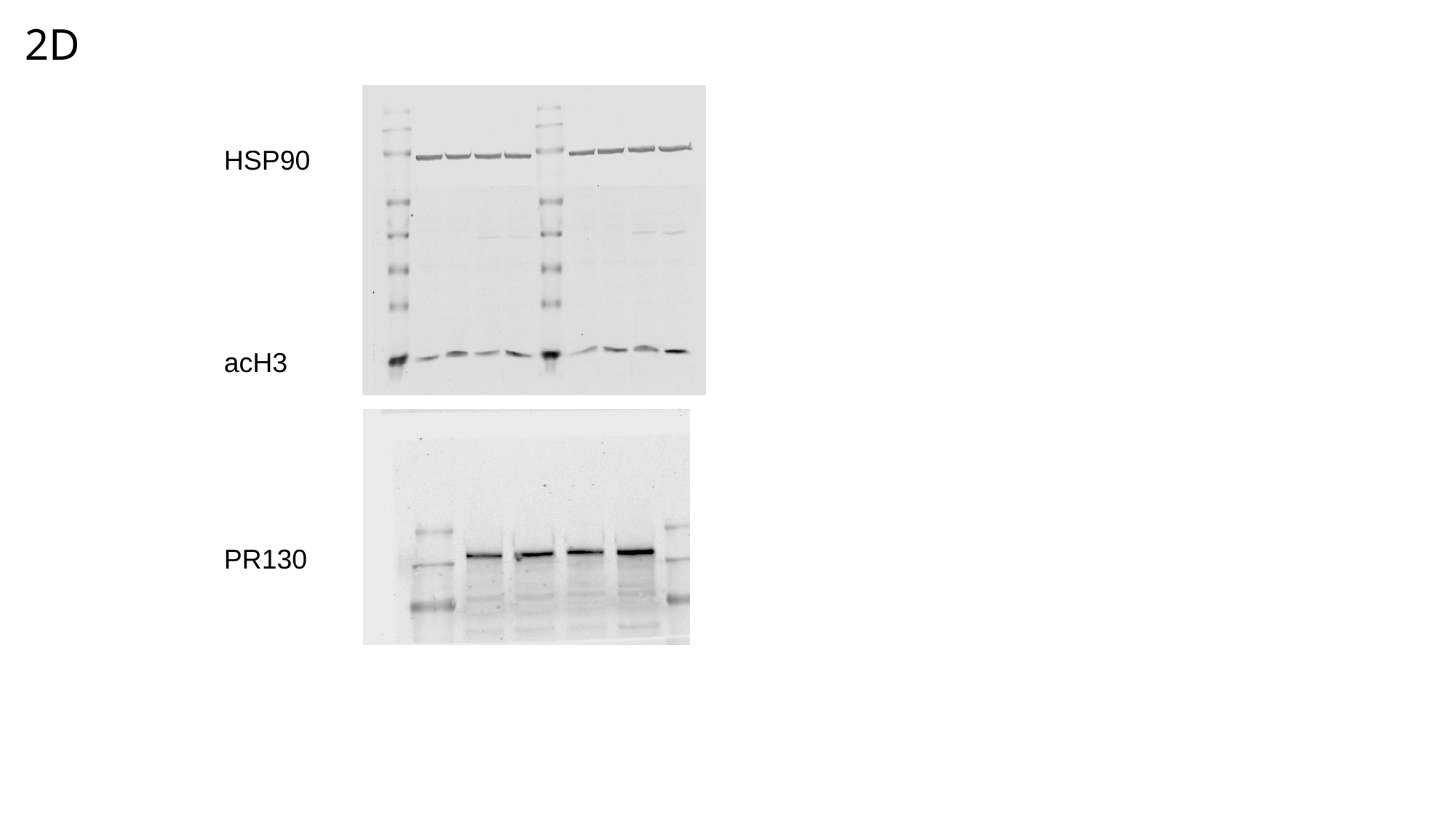

2D
HSP90
acH3
PR130

## Slide 5
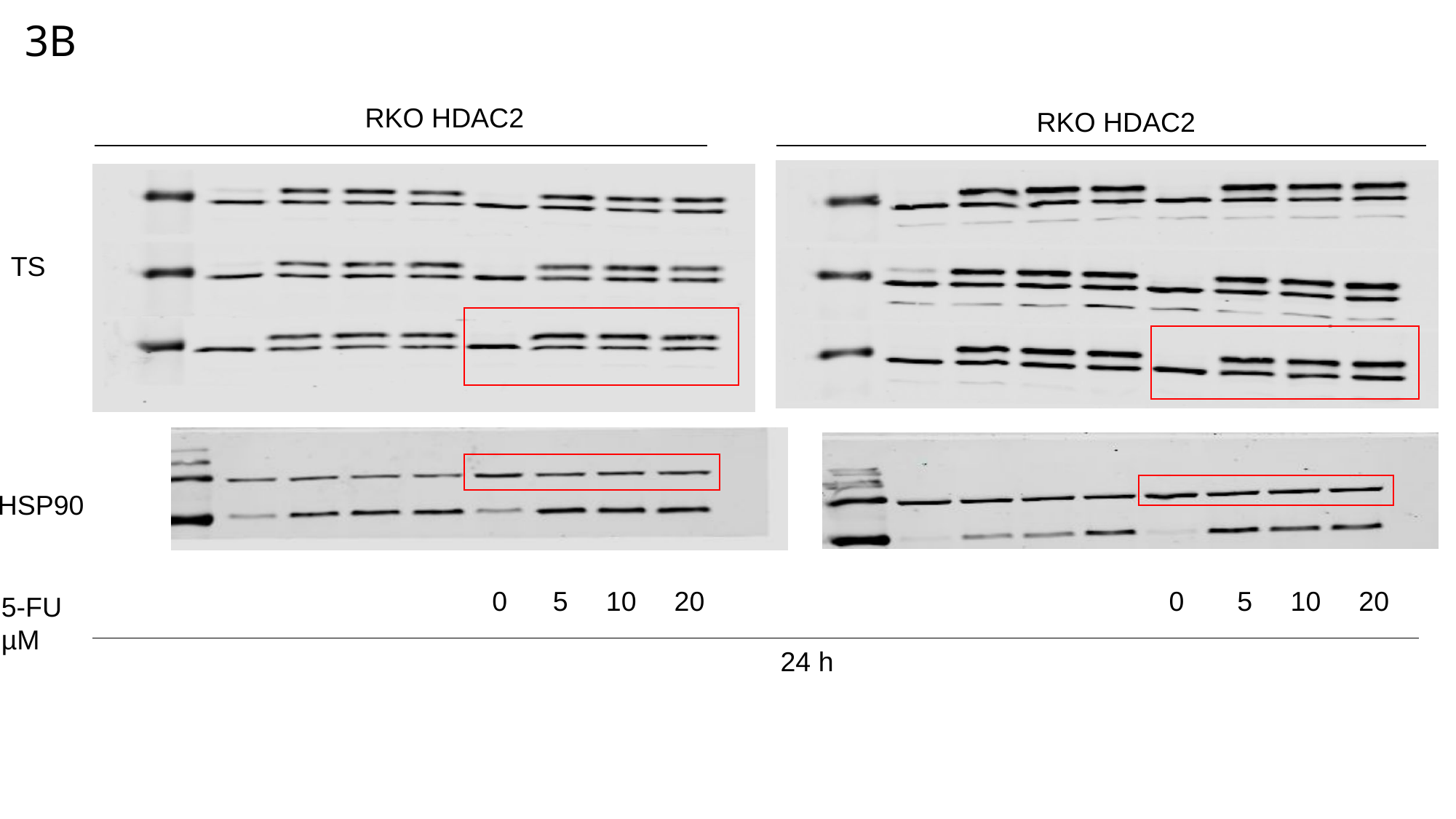

3B
RKO HDAC2
TS
HSP90
 0 5 10 20 0 5 10 20
5-FU
µM
24 h

## Slide 6
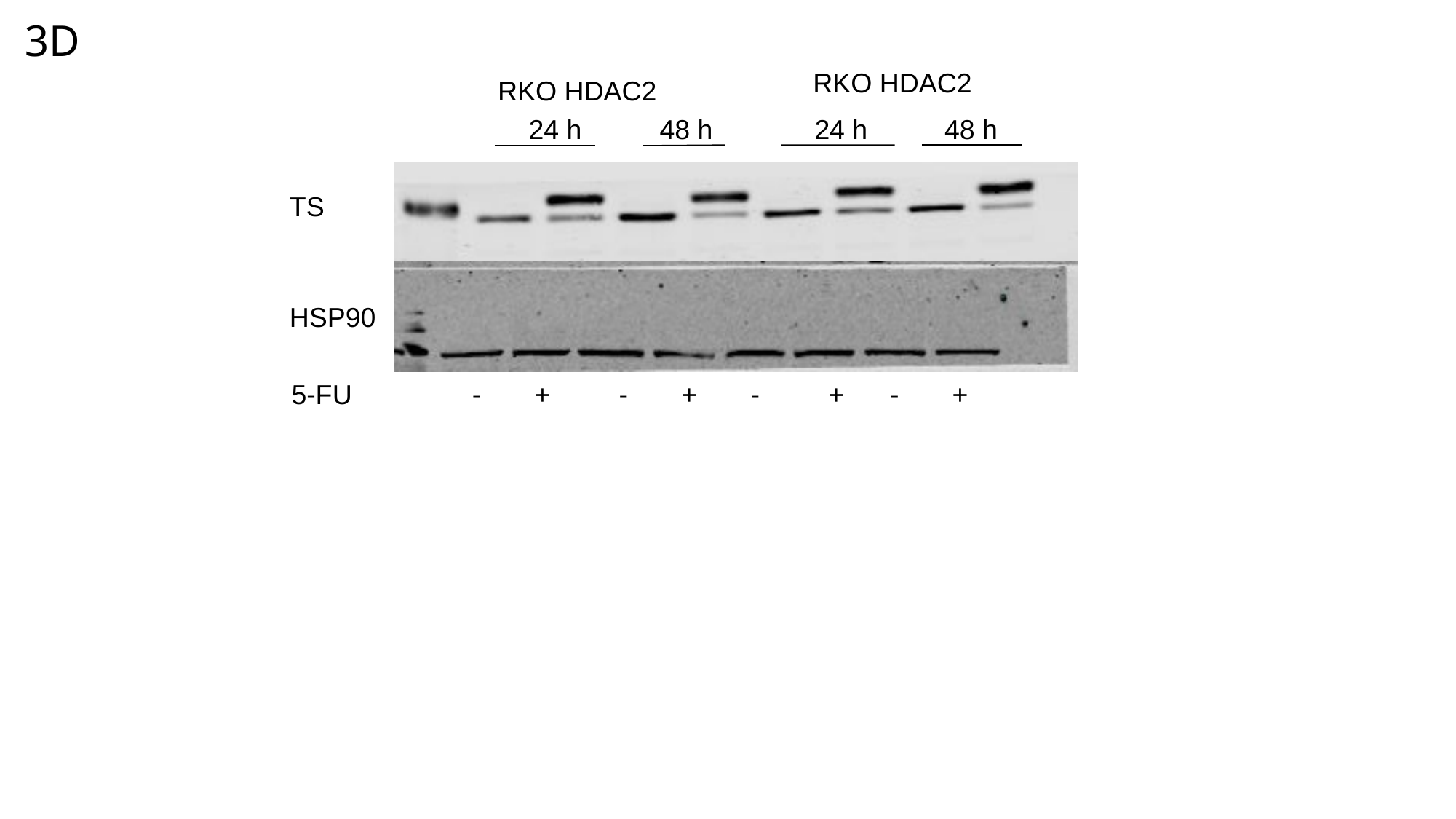

3D
RKO HDAC2
24 h
48 h
24 h
48 h
TS
HSP90
5-FU
 - + - + - + - +

## Slide 7
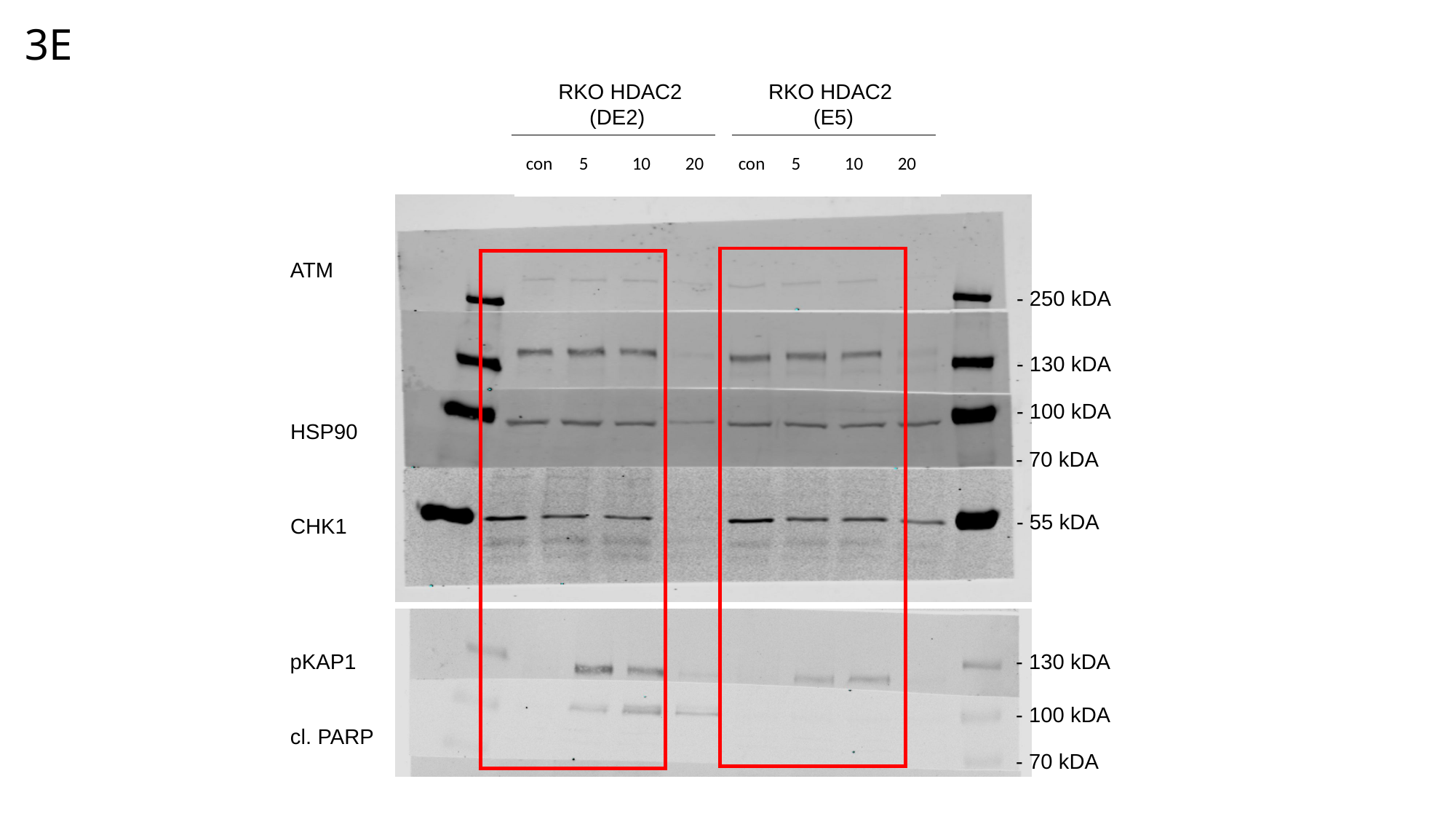

3E
RKO HDAC2
(E5)
| con | 5 | 10 | 20 | con | 5 | 10 | 20 |
| --- | --- | --- | --- | --- | --- | --- | --- |
ATM
- 250 kDA
- 130 kDA
- 100 kDA
HSP90
- 70 kDA
- 55 kDA
CHK1
pKAP1
- 130 kDA
- 100 kDA
cl. PARP
- 70 kDA

## Slide 8
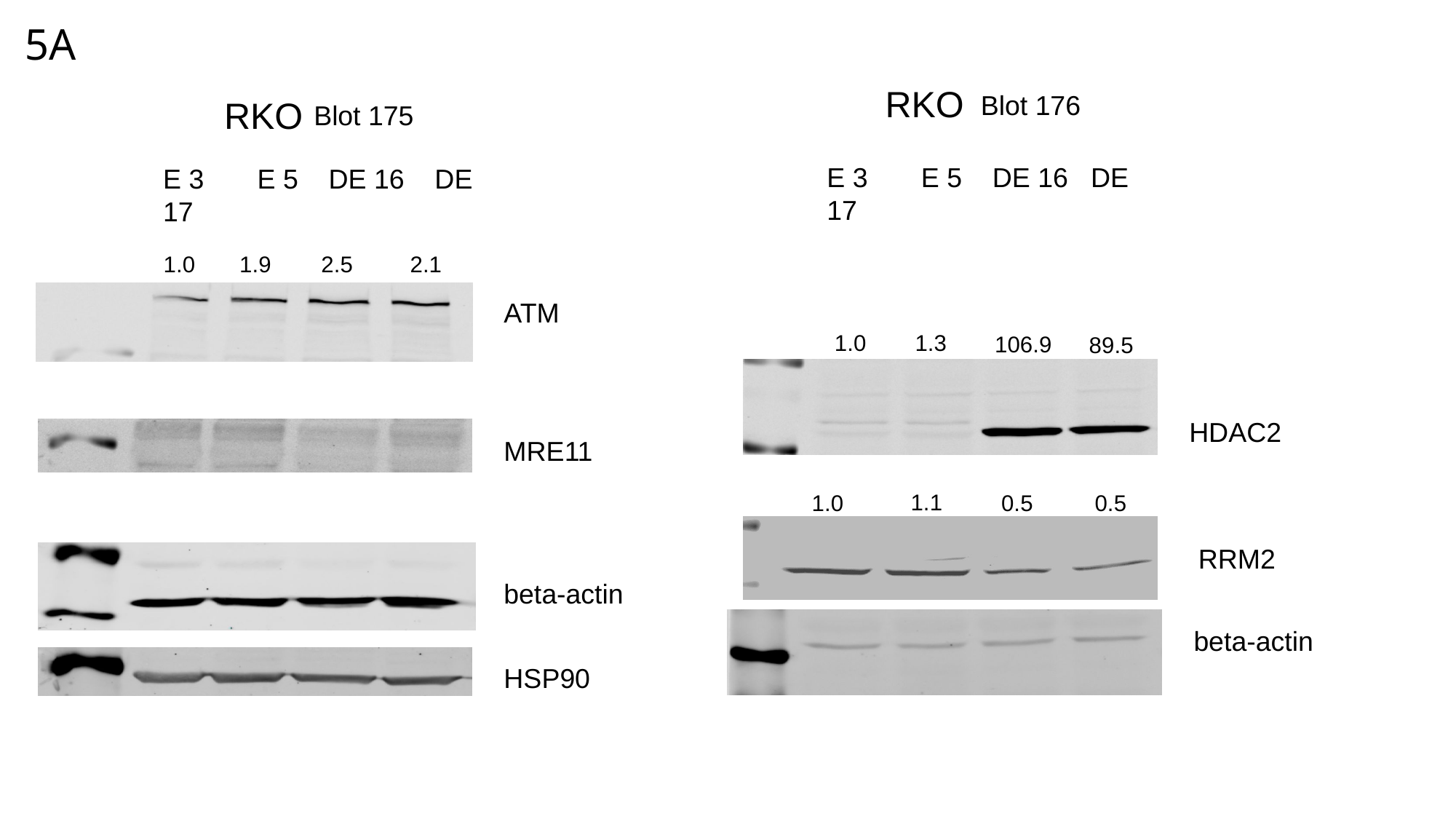

5A
RKO
Blot 176
RKO
Blot 175
E 3 E 5 DE 16 DE 17
E 3 E 5 DE 16 DE 17
1.0
2.1
2.5
1.9
ATM
1.0
1.3
106.9
89.5
HDAC2
MRE11
1.1
0.5
0.5
1.0
RRM2
beta-actin
beta-actin
HSP90

## Slide 9
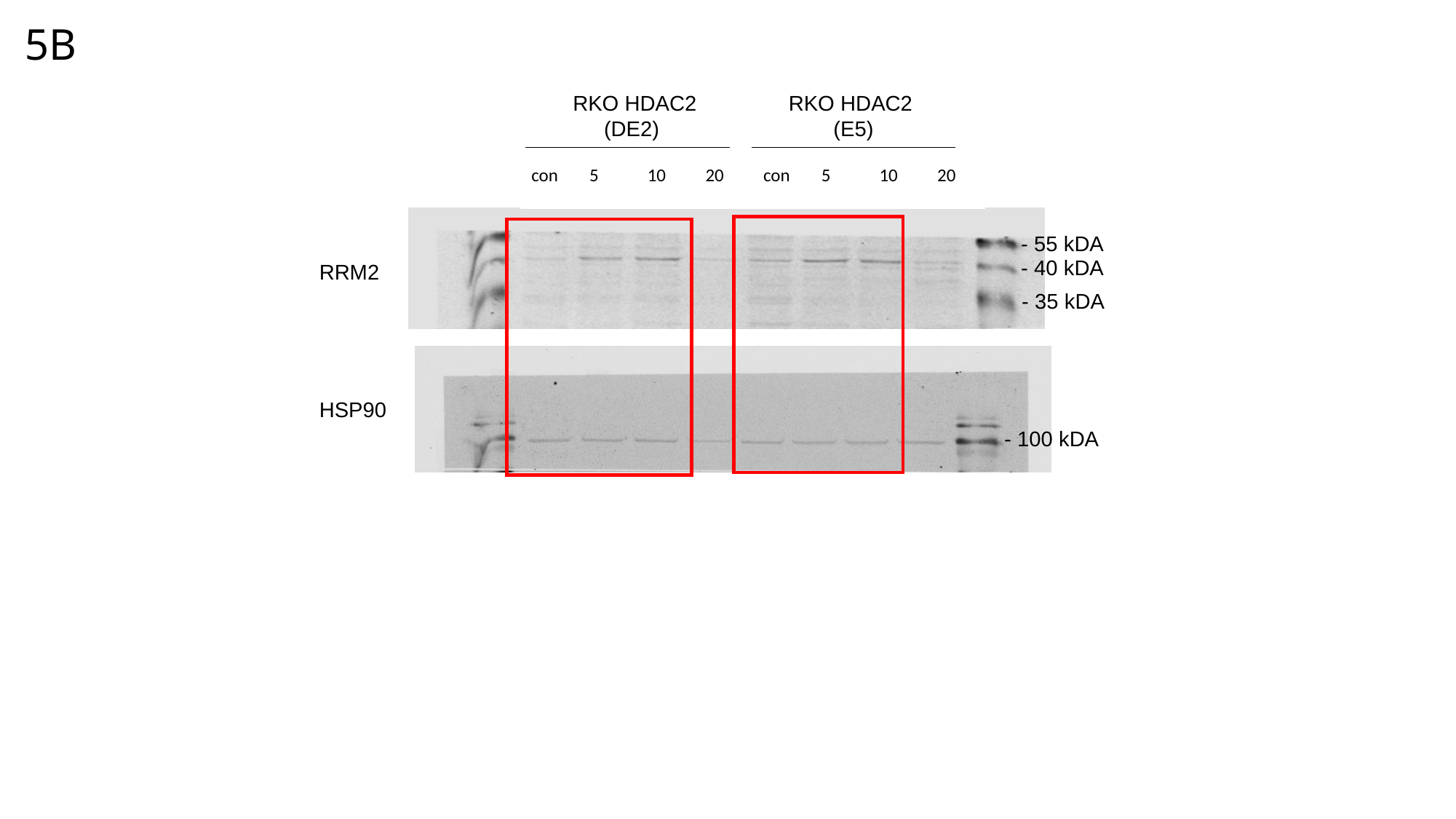

5B
RKO HDAC2
(E5)
| con | 5 | 10 | 20 | con | 5 | 10 | 20 |
| --- | --- | --- | --- | --- | --- | --- | --- |
- 55 kDA
- 40 kDA
RRM2
- 35 kDA
HSP90
- 100 kDA

## Slide 10
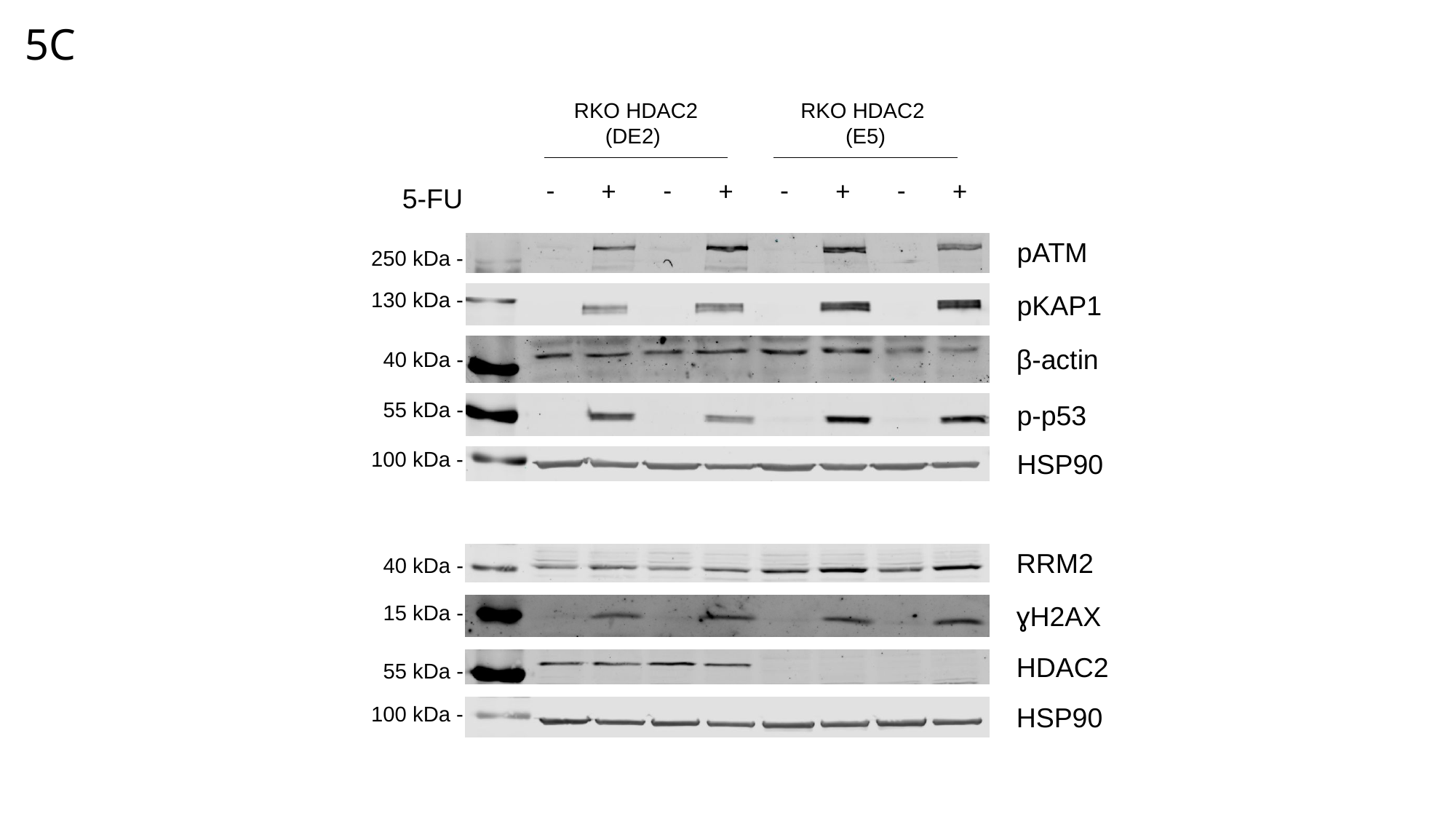

5C
RKO HDAC2
(DE2)
| - | + | - | + | - | + | - | + |
| --- | --- | --- | --- | --- | --- | --- | --- |
5-FU
pATM
250 kDa -
130 kDa -
pKAP1
β-actin
40 kDa -
55 kDa -
p-p53
100 kDa -
HSP90
RRM2
40 kDa -
ɣH2AX
15 kDa -
HDAC2
55 kDa -
100 kDa -
HSP90

## Slide 11
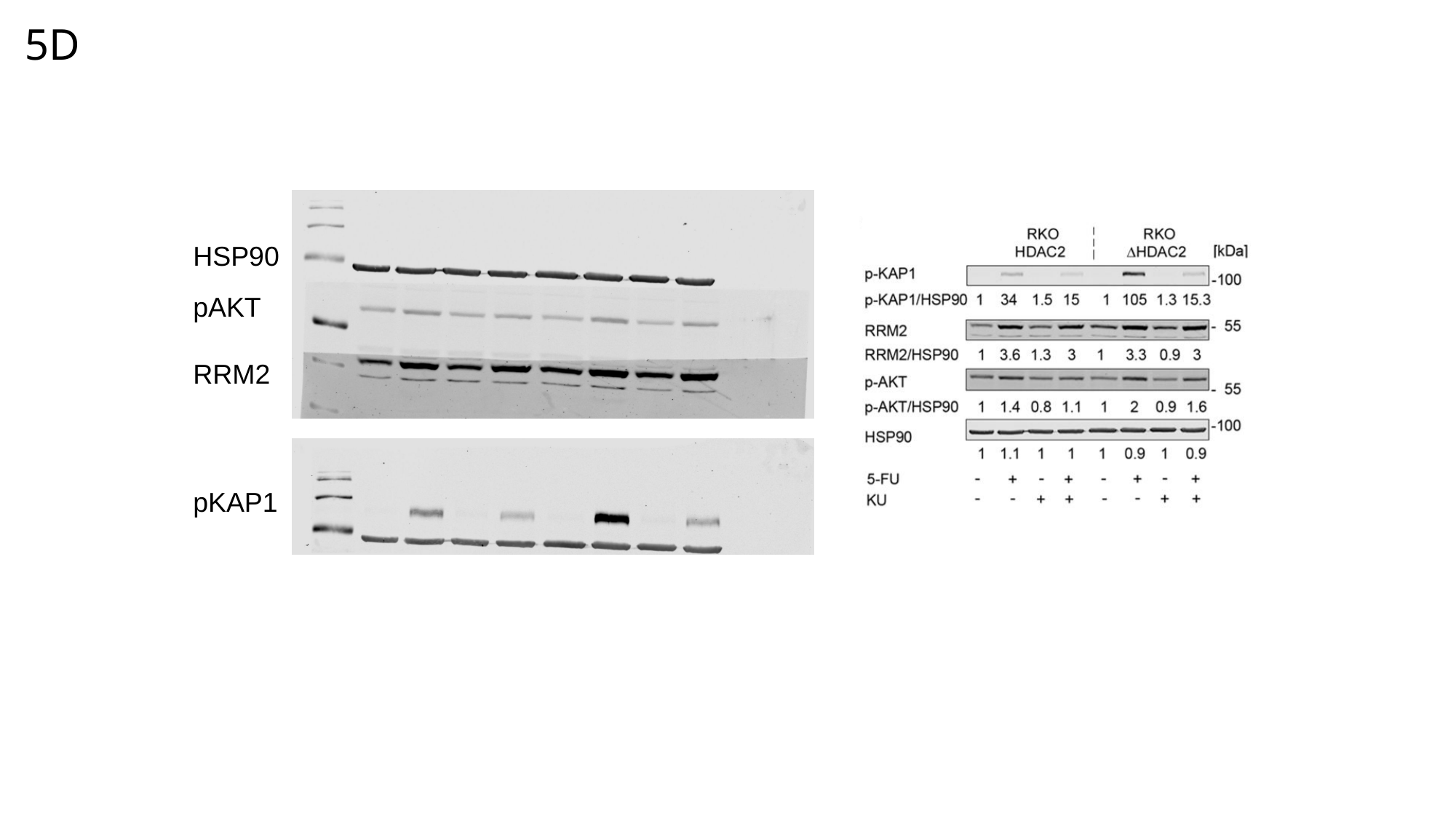

5D
HSP90
pAKT
RRM2
pKAP1
