# Supplementary material for: The epigenetic modifier HDAC2 and the checkpoint kinase ATM determine the responses of microsatellite instable colorectal cancer cells to 5-fluorouracil
Source: Cell Biol Toxicol. 2022 May 24;39(5):2401–19. doi: 10.1007/s10565-022-09731-3 (PMC10547618; doi:10.1007/s10565-022-09731-3)
Supplement: Supplementary file 3 — Supplementary file3 (DOCX 24 KB) [file 10565_2022_9731_MOESM3_ESM.docx]

| **MMR-Status** | MSI-H | MSI-H | MSI-H | MSI-H | MSI-H |  |  | **Supplementary** **Table S1 Allelic list of STR analysis.** DNA fingerprinting analysis of the RKO ATCC (RKO), RKO-ES (RKO ES), RKO HDAC2 positive (RKO HDAC2 pos.), and the RKO HDAC2 negative (RKO HDAC2 neg.) cell lines using 8 different, highly polymorphic short tandem repeat (STR) loci. Generated STR profiles were compared to a parental reference profile of the cell line RKO (ATCC (CRL-2577)) as indicated by a search of the databases of cell banks ATCC (USA), JCRB (Japan), RIKEN (Japan), KCLB (Korea) and DSMZ (Germany) and thereby confirmed to be authentic RKO cell cultures. The analysis was conducted by the Leibniz-Institute DSMZ (Braunschweig, Germany). Samples were tested negative for presence of mitochondrial DNA form rodent cells as mouse (M), rat (R), Chinese (CH) and Syrian (SH) hamster and were confirmed to show drifted or lost alleles at STR loci due to Microsatellite Instability (MSI) upon deficiency in DNA mismatch repair. |
| --- | --- | --- | --- | --- | --- | --- | --- | --- |
| **SH** |  | - | - | - | - |  |  |  |
| **CH** |  | - | - | - | - |  |  |  |
| **R** |  | - | - | - | - |  |  |  |
| **M** |  | - | - | - | - |  |  |  |
| **Date Animal-PCR:** |  | 31.07.2017 | 31.07.2017 | 31.07.2017 | 31.07.2017 |  |  |  |
| **Amel'** | X | X | X | X | X |  |  |  |
| **Amel** | X | X | X | X | X |  |  |  |
| **CSF1'** | 10 | 11 | 11 | 11 | 11 |  |  |  |
| **CSF1** | 8 | 8 | 8 | 8 | 8 |  |  |  |
| **TPOX'** | 11 | 11 | 11 | 11 | 11 |  |  |  |
| **TPOX** | 11 | 10 | 10 | 10 | 10 |  |  |  |
| **TH01'** | 10 | 9 | 10 | 8 | 9 |  |  |  |
| **TH01** | 6 | 6 | 6 | 6 | 6 |  |  |  |
| **vWA'** | 17 | 22 / 23 | 19 / 22/ 23/ 24 | 19 / 23/ 24 | 19 / 22 / 23 |  |  |  |
| **vWA** | 15 / 16 | 16 | 16 / 17 | 16 | 16 |  |  |  |
| **D16'** | 13 | 13,1 | 13 | 13,1 | 13,1 |  |  |  |
| **D16** | 12 | 12 / 13 | 12 | 12 / 13 | 12 / 13 |  |  |  |
| **D7'** | 10 | 10 | 11 | 10 | 10 |  |  |  |
| **D7** | 8 | 8 | 8 | 8 | 8 |  |  |  |
| **D13'** | 11 | 11 | 11 | 12 | 11 |  |  |  |
| **D13** | 8 | 8 | 8 | 8 | 8 |  |  |  |
| **D5'** | 13 | 13 | 12 | 13 | 13 | STR-profile of reference | STR-profile of analysed sample |  |
| **D5** | 11 | 11 | 11 | 11 | 11 |  |  |  |
| **Date** | 02.09.2011 | 31.07.2017 | 31.07.2017 | 31.07.2017 | 31.07.2017 |  |  |  |
| **Cell line** | RKO | RKO | RKO ES | RKO HDAC2 pos | RKO HDAC2 neg |  |  |  |
|  | ATCC (CRL-2577) | Krämer; Universitätsmedizin Mainz; Institut für Toxikologie | Krämer; Universitätsmedizin Mainz; Institut für Toxikologie | Krämer; Universitätsmedizin Mainz; Institut für Toxikologie | Krämer; Universitätsmedizin Mainz; Institut für Toxikologie |  |  |  |
