# Supplementary material for: The epigenetic modifier HDAC2 and the checkpoint kinase ATM determine the responses of microsatellite instable colorectal cancer cells to 5-fluorouracil
Source: Cell Biol Toxicol. 2022 May 24;39(5):2401–19. doi: 10.1007/s10565-022-09731-3 (PMC10547618; doi:10.1007/s10565-022-09731-3)
Supplement: Supplementary file 4 — Supplementary file4 (DOCX 140 KB) [file 10565_2022_9731_MOESM4_ESM.docx]

| ID | Sample Type | Type | Site | T | N | Lymph Nodes | | M | G | R | L | V | UICC | Sex | Age | Therapy | | Recurrence | Secondary Therapy | | RFS | OS | Mol. Subtype | P53 | K-Ras | B-Raf | MSI |
| --- | --- | --- | --- | --- | --- | --- | --- | --- | --- | --- | --- | --- | --- | --- | --- | --- | --- | --- | --- | --- | --- | --- | --- | --- | --- | --- | --- |
|  |  |  |  |  |  | 🞤 | ∑ |  |  |  |  |  |  |  |  | Surgery | Chemo |  | Intention | Treatment |  |  |  |  |  |  |  |
| HROC24 | Carcinoma | Adenocarcinoma | Right colon | T2 | N0 | 0 | 13 | M0 | G2 | R0 | L0 | V1 | UICC I | M | 98 | c |  |  |  |  | progressive disease | | spMSI-H |  | wt | mut | MSI-H |
| HROC29 | Carcinoma | Adenocarcinoma | Right colon | T3 | N2 | 8 | 30 | M1 | G3 | R0 | L0 | V1 | UICC IV | M | 59 | c |  |  |  |  | progressive disease | | Lynch | wt | wt | wt | MSI-H |
| HROC48 | Carcinoma | Adenocarcinoma | Transverse | T2 | N1 | 3 | 39 | M0 | G3 | R2 | L1 | V0 | UICC III | F | 68 | c | c | m | c | s+ch | 55 | 133 | spMSI-H | wt | wt | wt | MSI-H |
| HROC48_Met | Metastasis |  | Peritoneum |  |  |  |  |  |  |  |  |  |  | F | 72 |  |  |  |  |  | 77 |  | spMSI-H | wt | wt | wt | MSI-H |
| HROC50_Tu1 | Carcinoma | Adenocarcinoma | Right colon | T4 | N0 | 0 | 34 | M0 | G2 | R0 | L0 | V0 | UICC II | F | 67 | c | c |  |  |  | 69 | F/U until 2016 | spMSI-H |  | wt | mut | MSI-H |
| HROC53 | Carcinoma | Adenocarcinoma | Right colon | T3 | N0 | 0 | 20 | M0 | G3 | R0 | L0 | V0 | UICC II | F | 72 | c |  |  |  |  | 135 | 135 | spMSI-H | wt | wt | wt | MSI-H |
|  |  |  |  |  |  |  |  |  |  |  |  |  |  |  |  |  |  |  |  |  |  |  |  |  |  |  |  |
|  |  |  |  |  |  |  |  |  |  |  |  |  |  |  |  | c = curative |  | m = metastasis |  | s = surgery |  |  |  |  |  |  |  |
|  |  |  |  |  |  |  |  |  |  |  |  |  |  |  |  |  |  |  |  | ch = chemo |  |  |  |  |  |  |  |
